# Trithorax dependent changes in chromatin landscape at enhancer and promoter regions drive female puberty

**DOI:** 10.1038/s41467-017-02512-1

**Published:** 2018-01-04

**Authors:** Carlos A. Toro, Hollis Wright, Carlos F. Aylwin, Sergio R. Ojeda, Alejandro Lomniczi

**Affiliations:** 1 Primate Genetics Section/Division of Neuroscience, Oregon National Primate Research Center/Oregon Health and Science University, 505 NW 185th Ave, Beaverton, OR 97006 USA; 2 Division of Neuroscience, Oregon National Primate Research Center/Oregon Health and Science University, 505 NW 185th Ave, Beaverton, OR 97006 USA

## Abstract

Polycomb group (PcG) proteins control the timing of puberty by repressing the *Kiss1* gene in hypothalamic arcuate nucleus (ARC) neurons. Here we identify two members of the Trithorax group (TrxG) of modifiers, mixed-lineage leukemia 1 (MLL1), and 3 (MLL3), as central components of an activating epigenetic machinery that dynamically counteracts PcG repression. Preceding puberty, MLL1 changes the chromatin configuration at the promoters of *Kiss1* and *Tac3*, two genes required for puberty to occur, from repressive to permissive. Concomitantly, MLL3 institutes a chromatin structure that changes the functional status of a *Kiss1* enhancer from poised to active. RNAi-mediated, ARC-specific *Mll1* knockdown reduced *Kiss1* and *Tac3* expression, whereas CRISPR-Cas9-directed epigenome silencing of the *Kiss1* enhancer selectively reduced *Kiss1* activity. Both interventions delay puberty and disrupt reproductive cyclicity. Our results demonstrate that an epigenetic switch from transcriptional repression to activation is crucial to the regulatory mechanism controlling the timing of mammalian puberty.

## Abstract (web summary)

Before the onset of puberty, Polycomb proteins repress the expression of *Kiss1* in KNDy neurons of the arcuate nucleus. Here, by CRISPR-Cas9-directed epigenome editing and RNAi, the authors show that coordinated action of Mll proteins at the *Kiss1* promoter and enhancer is required for correct timing of puberty.

## Introduction

The first endocrine manifestation of the initiation of puberty is a diurnal increase in pulsatile luteinizing hormone (LH) secretion from the pituitary gland^1^. This change is driven by an increase in episodic gonadotropin hormone-releasing hormone (GnRH) release from neurosecretory hypothalamic neurons. A primary transsynaptic mechanism underlying pulsatile GnRH release involves a specialized subset of neurons located in the arcuate nucleus (ARC) of the medial basal hypothalamus (MBH)^2,3^. They have been termed KNDy neurons, because they produce kisspeptin, neurokinin B (NKB) and dynorphin^2,4^. KNDy neurons release NKB, which acts on other KNDy neurons via specific receptors to stimulate kisspeptin release^2,4^. NKB and kisspeptin are released periodically, an oscillatory behavior determined by a phase-delayed inhibitory feedback of dynorphin on NKB release^2,4^. It is now clear that this periodicity underlies pulsatile LH release^5^, and that both kisspeptin and NKB are required for puberty to occur. Inactivating mutations of either *Kiss1* (encoding kisspeptin) or *Tac3* (encoding NKB) result in hypogonadotropic hypogonadism and pubertal failure in humans^6,7^. Although there is a population of kisspeptin neurons located in the anteroventral periventricular nucleus (AVPV) of rodents [reviewed in ref. ^8^], they do not contribute to the control of pulsatile GnRH release. Instead, AVPV neurons are required for the preovulatory surge of gonadotropins^8^. Accordingly, they do not appear to be involved in the initiation of female puberty, because the gonadotropin surge occurs only after the pubertal process is well underway.

In females, the secretory activity of KNDy neurons is kept in check during prepubertal development by an epigenetic mechanism of transcriptional repression^9^, exerted by the Polycomb group (PcG) of transcriptional silencers^10^. PcG proteins repress the *Kiss1* gene to prevent the premature initiation of the pubertal process^9^, and this effect takes place against a backdrop of repressive chromatin, that is, rich in histone modifications associated with gene repression (such as H3K27me3 and H3K9me3) and depleted of histone modifications associated with gene activation (such as H3K4me2/3 and H3K9,14ac)^11^. During embryonic development, PcG-dependent gene repression is counterbalanced by the Trithorax group (TrxG) of transcriptional activators. Together, PcG and TrxG proteins play a major, evolutionarily conserved, role in the epigenetic control of gene expression required for the establishment of cellular diversity^12^. TrxG proteins counteract the effect of PcG proteins by methylating histone 3 at lysine 4 (H3K4)^13,14^, a modification that establishes a permissive chromatin configuration, i.e., enriched in H3K4me2/3 and H3K9,14ac, with or without loss of H3K27me3 and H3K9me3^15,16^. In mammals, there are six TrxG protein complexes termed COMPASS (Complex of Proteins associated to Set1) and COMPASS-like, because they are related to the original yeast SET1 methyltransferase^13,14^. Two of these complexes (SET1A and SET1B COMPASS) contain *Drosophila* SET1-related proteins; two (known as COMPASS-like) contain the proteins MLL1/KMT2A or MLL2/KMT2B, which are related to *Drosophila* Trithorax (Trx), and the other two (also termed COMPASS-like) contain either MLL3/KMT2C or MLL4/KMT2D, both of them related to *Drosophila* Trr (Trx-related). While SET1A/SET1B and MLL1/MLL2 mediate H3K4 trimethylation (H3K4me3) at promoters^17,18^, MLL3/MLL4 catalyze monomethylation of H3K4 (H3K4me1) at enhancer sites^19^.

It has been shown that as the PcG repressive influence wanes before puberty, the abundance of histone modifications either catalyzed by (H3K4me3), or associated to (H3K9,14ac) the Trithorax group (TrxG) of transcriptional activators^13^ increases at the *Kiss1* promoter^9^, implicating the TrxG complex as the facilitatory counterpart of PcG-mediated gene silencing. Should these interactions also occur at the *Tac3* promoter, it would strongly suggest that the epigenetic control of KNDy neurons, and hence the pubertal process itself, is a coordinated process involving the two principal gene systems that, operating within the ARC, spur reproductive development. The presence of both repressive and stimulatory epigenetic pathways regulating *Kiss1* and *Tac3* expression in KNDy neurons suggests that a switch from epigenetic repression to activation within these neurons underlies the developmental process by which GnRH release increases by late juvenile development to bring about the pubertal process. In a broader context, it also raises the tantalizing possibility that mammalian puberty might be a major developmental milestone regulated by the counterbalancing actions of the PcG and TrxG complexes operating within the neuroendocrine brain. The present study provides experimental evidence supporting this concept.

## Results

### TrxG gene expression in the MBH during pubertal development

**Fig. 1:**
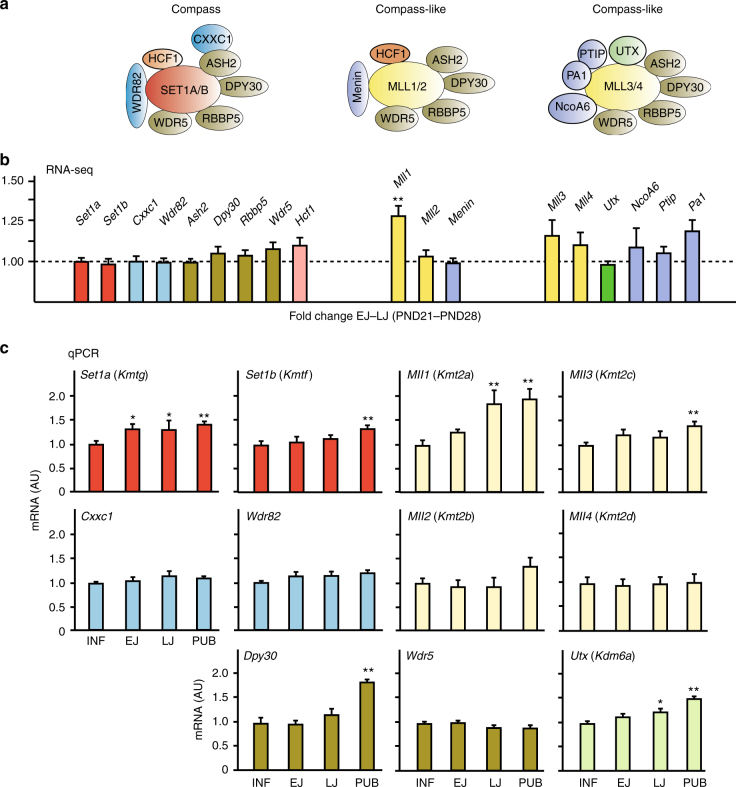
TrxG gene expression in the prepubertal female rat hypothalamus. **a** The COMPASS families (for details see Introduction). **b** Expression of COMPASS genes in the MBH of prepubertal female rats assessed by RNA-seq. Data are represented as fold change between the early juvenile and the late juvenile groups (EJ/LJ). Dotted line depicts the EJ/LJ ratio of 1. Bars represent mean ±s.e.m. (*n* = 4). **P < 0.01 vs. EJ group; Student’s *t*-test. **c** Expression of COMPASS genes in the MBH of peripubertal female rats as determined by qPCR. RNA expression data were normalized using peptidylprolyl isomerase A as the housekeeping gene and by dividing each individual value by the average of the INF group. Bars represent mean ±s.e.m. INF = infantile (*n* = 7–8); EJ = early juvenile (*n* = 5–7); LJ = late juvenile (*n* = 6–8); PUB = pubertal (*n* = 6–9), day of the first preovulatory surge of gonadotropins. *P < 0.05; and **P < 0.01 vs. INF; one-way ANOVA followed by Student–Newman–Keuls (SNK) test

**Fig. 2:**
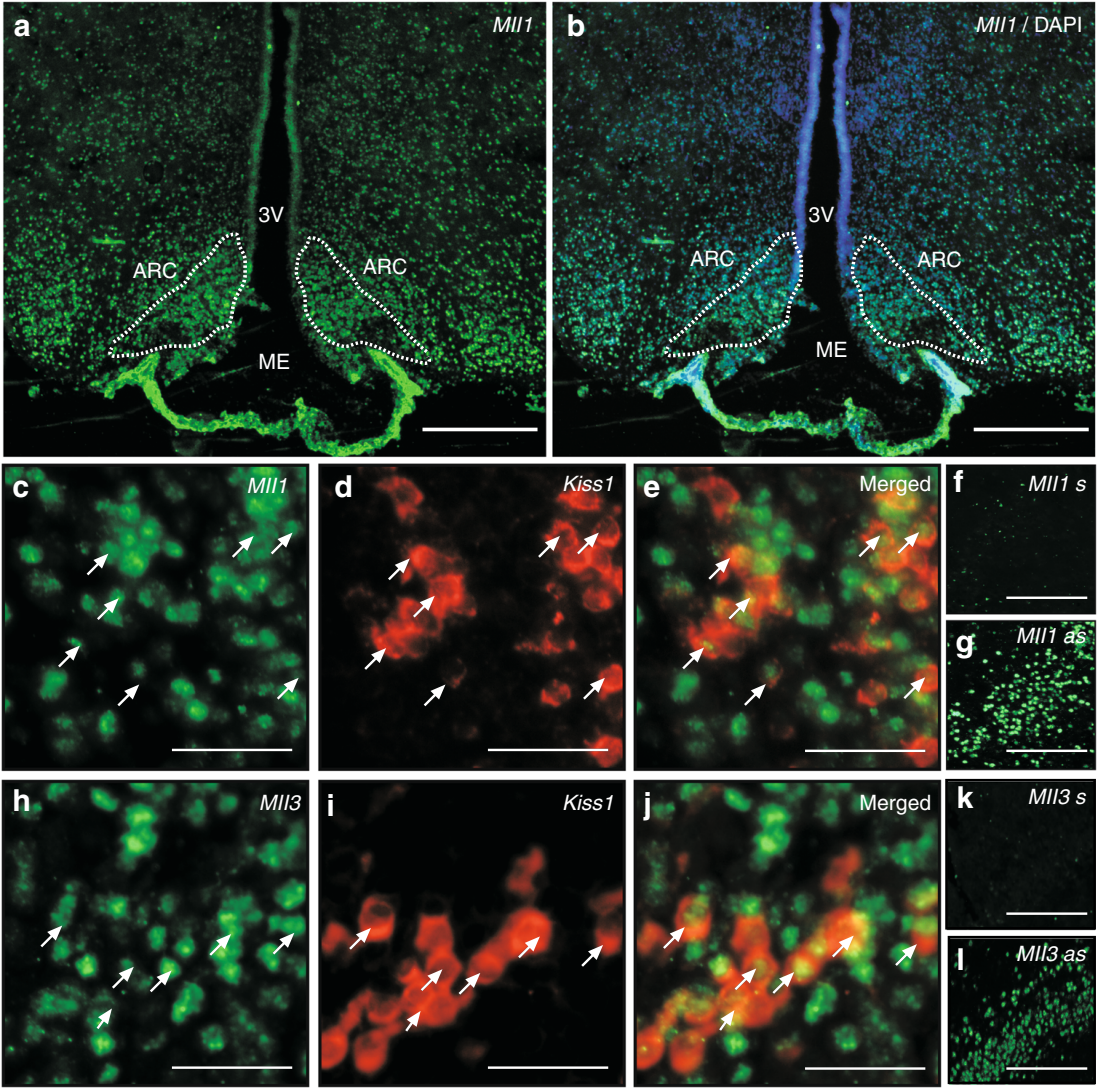
*Mll1* and *Mll3* mRNA transcripts in KNDy neurons of late juvenile female rats. **a**, **b** Abundance of *Mll1* mRNA transcripts (green color) in the arcuate nucleus (ARC, dotted line) region of the MBH, as determined by FISH. 3V, third ventricle; ME, median eminence. Bars, 500 µm. **c**–**e** KNDy neurons of the ARC contain *Mll1* mRNA as determined by double FISH (*Kiss1* mRNA, red; *Mll1* mRNA, green). **h**–**j** KNDy neurons also contain *Mll3* mRNA. Bars, 100 µm. **f**, **g** Lack of hybridization in sections incubated with a sense * Mll1 * RNA probe. **k**, **l** Lack of hybridization in sections incubated with a sense *Mll3* RNA probe. Bars, 200 µm. s = sense; as = antisense

We used massively parallel sequencing (RNA-seq) to determine if the advent of female puberty is preceded by changes in MBH expression of any of the 18 genes known to be components of the TrxG complex. We examined these changes in female rats during juvenile development (postnatal days (PND) 21–28), i.e., at the time when expression of *Eed* and *Cbx7*, two key members of the PcG complex, declines^9^. Only *Mll1*, a member of one of the COMPASS-like family of TrxG genes^12^ (Fig. 1a), showed increased expression between the beginning (EJ/PND21) and the end (LJ/PND28) of the juvenile period (Fig. 1b). A more detailed quantitative PCR (qPCR) examination of TrxG gene expression before and at the completion of puberty revealed that *Mll1* mRNA levels remain elevated on the day when the first preovulatory surge of gonadotropins takes place (Fig. 1c), i.e., at the completion of puberty. Though less noticeable, expression of other TrxG genes also increases either before (*Set1a* and *Utx*) or at the time of puberty (*Set1b*, *Mll3*, and *Dpy30*). *Set1a* and *Set1b* encode methyltransferases responsible for the bulk di- and trimethylation of lysine 4 at histone 3 across the genome^13,14^. The MBH from female rhesus monkeys undergoing puberty exhibited an increase in expression of *MLL1*, *SET1A*, and *DPY30* similar to that detected in rats (Supplementary Fig. 1), indicating that puberty-related changes in hypothalamic TrxG expression are not limited to rodents, but also occur in nonhuman primates. Using double fluorescent in situ hybridization (FISH) we observed that cells expressing *Mll1* mRNA were abundant in the ARC (Fig. 2a, b) and that within this region KNDy neurons, identified by the presence of *Kiss1* mRNA, contain *Mll1* mRNA transcripts (Fig. 2c–e). KNDy neurons also express *Mll3* (Fig. 2h–j), a member of the COMPASS family that activates distal enhancer sites by monomethylating histone H3K4, promoting H3K27 acetylation, and antagonizing PcG silencing^19,20^. In addition, KNDy neurons express other key components of the TrxG complex, including *Set1b* (Supplementary Fig. 2), *Mll2* (Supplementary Fig. 3), and *Mll4* (Supplementary Fig. 4). MLL4 stimulates enhancer activity by eliciting changes in histone configuration similar to those catalyzed by MLL3^19,20^. These results demonstrate that KNDy neurons express all members of the TrxG activating complex required to counteract PcG silencing, and epigenetically enhance the transcription of genes involved in the stimulatory control of puberty.

### Recruitment of TrxG proteins to puberty-activating genes

We next used chromatin immunoprecipitation (ChIP)-qPCR assays to determine if recruitment of core COMPASS-like proteins to the promoter region of *Kiss1*, *Tac3*, and *Pdyn*, the genetic backbone of KNDy neuron reproductive function, changes in the MBH during the days antedating puberty. We focused our attention to COMPASS-like complexes containing MLL1–MLL4 instead of SET1A or SET1B, because MLL1–MLL4-dependent H3K4 methylation is an event that affects a selected population of genes involved in regulating discrete and specialized functions, in contrast to SET1A and SET1B, which are responsible for the global, genome-wide, di- and trimethylation of H3K4^13,14^. Regulation of *Kiss1* and *Tac3* gene expression in the ARC by TrxG proteins in the context of pubertal development is, undoubtedly, one of these specialized processes.

**Fig. 3:**
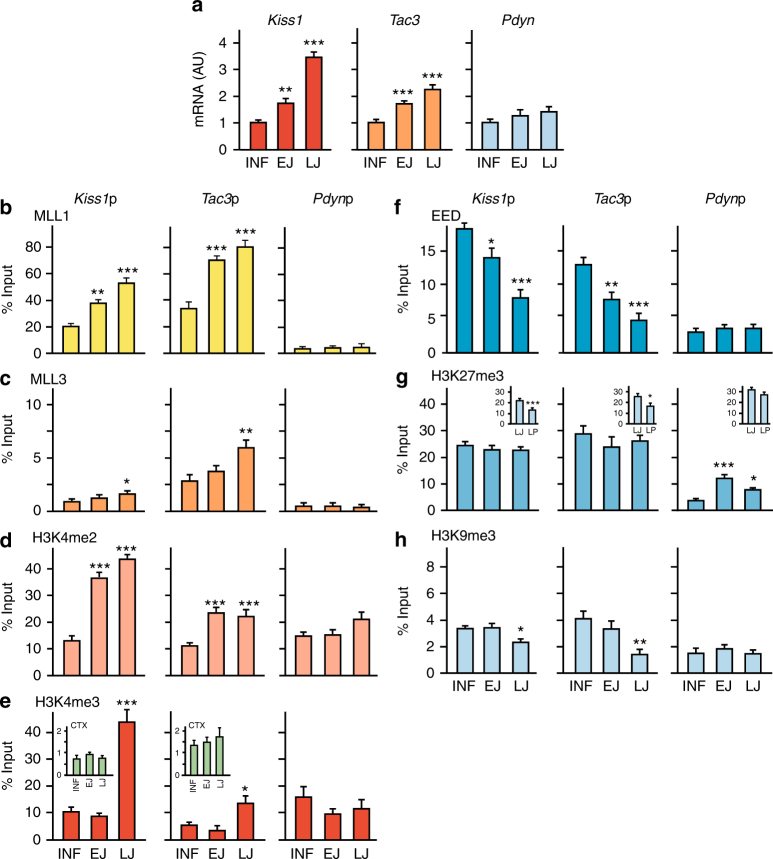
*Kiss1*, *Tac3*, and *Pdyn* mRNA expression, and promoter chromatin state in the prepubertal female rat hypothalamus. **a** Expression of *Kiss1*, *Tac3*, and *Pdyn* mRNA in the MBH of prepubertal female rats as determined by qPCR. Bars represent mean ±s.e.m. (INF, EJ, and LJ; *n* = 8); AU = arbitrary units. RNA expression data were normalized by dividing each individual value by the average of the INF group. **b** Changes in MLL1 recruitment to the *Kiss1*, *Tac3*, and Pdyn promoters before puberty. **c** Changes in MLL3 recruitment to the *Kiss1*, *Tac3*, and Pdyn promoters before puberty. **d**, **e** Abundance of the TrxG-dependent activating marks H3K4me2 (**d**) and H3K4me3 (**e**) during prepubertal development. Insets in **e** depict H3K4me3 content at the *Kiss1* and *Tac3* promoters in the CTX during prepubertal development. **f** EED recruitment to the *Kiss1*, *Tac3*, and *Pdyn* promoters before puberty. **g** Abundance of the PcG-dependent repressive mark H3K27me3 at the *Kiss1*, *Tac3*, and *Pdyn* before puberty or at the time of the first preovulatory surge of gonadotropins (late proestrus, LP) at the *Kiss1* and *Tac3* promoters (insets in **g**). **h** Abundance of the PcG-independent repressive mark H3K9me3 at the *Kiss1*, *Tac3*, and *Pdyn* promoters before puberty. Bars represent mean ±s.e.m. (INF, EJ, and LJ; *n* = 5–8; insets in **e**; *n* = 4; insets in **g**; *n* = 6). *P < 0.05, **P < 0.01, and ***P < 0.001 vs. INF; one-way ANOVA followed by SNK test

Both *Kiss1* and *Tac3* mRNA levels begin to increase in the ARC at the initiation of juvenile development (EJ) with respect to the infantile phase (INF/PND14), becoming even more elevated by the LJ period, i.e., at the initiation of puberty (Fig. 3a). In contrast, *Pdyn* expression remained unchanged during the entire prepubertal period (Fig. 3a). Consistent with the concept of a coordinated epigenetic input regulating the stimulatory arm of KNDy neurons, we observed that the abundance of MLL1 at both the *Kiss1* and *Tac3* promoters increases concomitantly with the change in gene expression (Fig. 3b). MLL2 association to the *Kiss1* promoter did not change, but increased modestly at the *Tac3* promoter at LJ (Supplementary Fig. 5a). In contrast, recruitment of both MLL1 and MLL2 to the *Pdyn* promoter remained low and unaltered throughout the entire postnatal period studied (Fig. 3b and Supplementary Fig. 5a). Assessment of MLL3 recruitment revealed an increase in MLL3 association to both the *Kiss1* and *Tac3* promoters at LJ, with no changes detected at the *Pdyn* promoter (Fig. 3c). In contrast to MLL3, MLL4 association to each of these three promoters remained unaltered (Supplementary Fig. 5b). Concordant with the increase in MLL1 association to the *Kiss1* and *Tac3* promoters, H3K4me2 abundance at both promoters (but not at the *Pdyn* promoter) increased strikingly between the INF and EJ periods, remaining elevated thereafter (Fig. 3d). A similar strong increase in H3K4me3 content was observed at both the *Kiss1* and *Tac3* promoters, but instead of occurring at EJ, it became apparent at LJ, Fig. 3e), when puberty is initiated. These changes were hypothalamic-specific, because they were not observed in the cerebral cortex (CTX; insets in Fig. 3e). They were correlated with increases in both *Kiss1* and *Tac3* mRNA levels that began at EJ, when H3K4me2 content was elevated, and became maximal at LJ, coinciding with the prepubertal increase in H3K4me3 content (Fig. 3a).

In contrast to TrxG proteins and associated histone marks, EED—a PcG protein required for PcG-mediated gene silencing—was evicted from the *Kiss1* and *Tac3* promoters during juvenile development (EJ–LJ; Fig. 3f). As shown before^9^, depletion of H3K27me3 (the histone modification catalyzed by the PcG complex) did not occur at this time (Fig. 3g). Instead, H3K27me3 content decreased at the late proestrus (LP) stage (insets in Fig. 3g), the phase of puberty when the first preovulatory surge of gonadotropins takes place. The coexistence of the permissive/activating marks H3K4me2 and H3K4me3 with the repressive H3K27me3 modification is a feature of “bivalent” promoters (i.e., promoters of genes poised for activation in response to incoming stimuli)^21^. Based on these features, the *Kiss1* promoter has been postulated to be bivalent^9^.

Contrary to *Kiss1* and *Tac3*, H3K27me3 content at the *Pdyn* promoter increased during juvenile development (Fig. 3g). Although this change implies the institution of a PcG-dependent repression of *Pdyn* transcription at puberty, the lack of concomitant changes in EED recruitment to the *Pdyn* promoter (Fig. 3f) makes this inference less certain. Both the *Kiss1* and *Tac3* promoters appear to be subjected to some PcG-independent repressive control, as suggested by the mild loss of H3K9me3, a repressive mark deposited by the histone methyltransferase SETDB1^22^, from both the *Kiss1* and *Tac3* promoters at LJ (Fig. 3h). Overall, these results indicate that a dynamic balance between the PcG and TrxG complexes regulating *Kiss1* and *Tac3* expression in the ARC is a central feature of the epigenetic control of puberty

### MLL1 trans-activates puberty-activating genes

**Fig. 4:**
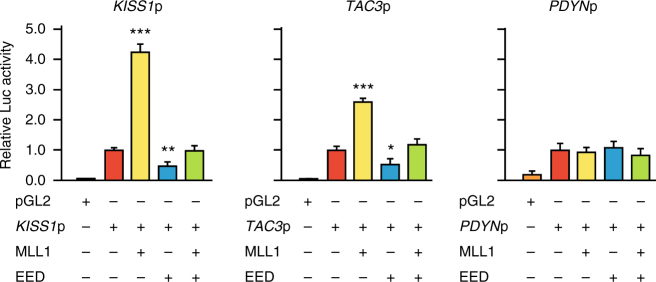
Effect of MLL1 and EED on *KISS1*, *TAC3*, and *PDYN* promoter (p) activity. Bars represent mean ± s.e.m. (*n* = 6). ***P < 0.001 vs. all other groups; **P < 0.01 vs. *Kiss1* promoter alone; *P < 0.05 vs. *Tac3* promoter alone; one-way ANOVA followed by SNK test

Consistent with this concept, MLL1 enhanced *Kiss1* and *Tac3*, but not *Pdyn*, transcriptional activity in gene promoter assays, and EED abolished this effect (Fig. 4). MLL1 also enhanced the transcriptional activity of *Ttf1*, *Eap1*, and *Nell2* (Supplementary Fig 6a), a set of genes involved in the stimulatory control of female puberty^23–25^, but did not increase the transcription of genes keeping the pubertal process in check (*Eed*, *MKRN3*, and *PENK*; Supplementary Fig 6b). For a trans-activating effect of MLL1 to also occur in vivo, MLL1 recruitment to these promoters would be expected to increase with the advent of puberty. Our results show that whereas MLL1 recruitment to the *Kiss1* and *Tac3* promoters does increase markedly at this time (Fig. 3b), it remains unchanged at the *Nell2* and *Ttf1* promoters (selected as representing puberty-activating genes other than *Kiss1* and *Tac3*; Supplementary Fig. 6c). Altogether, these findings demonstrate that MLL1 binding to the *Kiss1* and *Tac3* promoters increases selectively at puberty and activates transcription of these two puberty-activating genes, which are essential for puberty to occur.

### ARC-targeted loss of *Mll1* delays puberty

To determine if loss of *Mll1* would affect the timing of puberty we used an RNAi approach. We generated lentiviral (LV) constructs targeting *Mll1* mRNA via short hairpin (sh) RNAs inserted into the body of miRNA-30 and under the control of the U6 promoter (Supplementary Fig. 7a **)**. Transduction of the R22 rat hypothalamic cell line with these constructs demonstrated that two of the four shRNAs tested (sh2612 and sh12751) were effective in lowering *Mll1* mRNA levels (Supplementary Fig. 7b). Because the LV constructs employed encode an enhanced green fluorescent protein (eGFP) protein, we used this feature to isolate via fluorescent-activated cell sorting (FACS; Supplementary Fig. 7c) the cells transduced with the two effective shRNAs. qPCR analysis verified the effectiveness of both sh2612 and sh12751 in reducing *Mll1* expression (Supplementary Fig. 7d). Both shRNAs also reduced the levels of *Kiss1* and *Tac3* mRNA (Supplementary Fig. 7d), without affecting *Eed* and *Pdyn* mRNAs (Supplementary Fig. 7e), suggesting that as previously shown by the results of promoter assays, MLL1 trans-activates *Kiss1* and *Tac3* gene expression, but not expression of puberty-inhibitory genes.

**Fig. 5:**
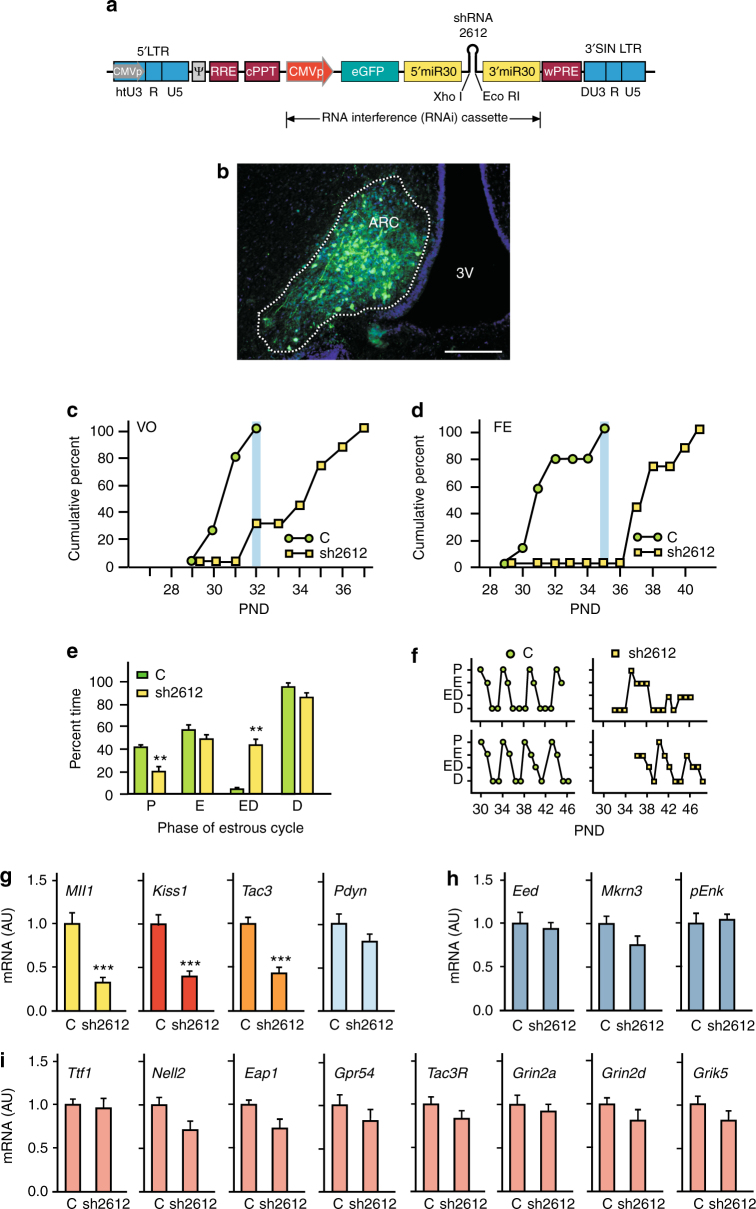
Silencing the *Mll1* gene in the ARC of immature female rats. **a** Lentiviral construct (LV-shMll1-GFP) encoding sh2612 that most effectively silences *Mll1*. **b** Transduction of ARC cells with sh2612 delivered to the ARC of EJ female rats. Green: sh2612 infected cells; blue: Hoechst-stained cell nuclei in the MBH; dotted line: ARC. Bars, 200 µm. **c** Cumulative percent day at vaginal opening (VO), and **d** cumulative percent day at first estrus (FE) in control (C; *n* = 9) and sh2612 (*n* = 7)-injected rats. Blue shade depicts the time when all C animals show VO or FE. **e** Percent of time spent in different stages of the estrous cycle by rats injected with C (*n* = 9) or sh2612 (*n* = 7). **f** Examples of estrous cycle patterns in rats injected with C or sh2612. **g** *Mll1*, *Kiss1*, *Tac3*, and *Pdyn* mRNA levels detected by qPCR in the ARC of LJ female rats receiving C (*n* = 8) or sh2612 (*n* = 8). **h** Expression of puberty-inhibitory or **i** puberty-activating genes in the ARC of LJ female rats receiving C (*n* = 8) or sh2612 (*n* = 8). RNA expression data were normalized by using peptidylprolyl isomerase A as the housekeeping gene and dividing each individual value by the average of the C group. *P* = proestrous; E = estrous; ED = transitional phase (estrous/diestrous); D = diestrous; AU = arbitrary units; 3V = third ventricle. Bars represent mean ±s.e.m. (**P < 0.01, ***P < 0.001 vs. C, Student’s *t*-test)

We then sought to determine if silencing of *Mll1* in the ARC is capable of delaying the time of puberty. We delivered an LV construct encoding shRNA 2612 (sh2612; Fig. 5a) to the ARC of EJ female rats via bilateral microinjections. Control animals were injected with an LV construct carrying GFP (C). Animals with correctly placed sh2612 (Fig. 5b) had a significant delay in both the age at vaginal opening (VO; Fig. 5c; mean age at VO: C, 31 ± 0.2 days vs. sh2612, 34.4 ± 0.7 days; *t* = 5.02, *P* < 0.001, Student’s *t*-test) and at first estrous (Fig. 5d); mean age at first estrous: C, 32 ± 0.6 days vs. sh2612, 38.3 ± 0.6 days; *t* = 7.25, *P* < 0.001, Student’s *t*-test). The first estrous is when the first ovulation takes place^9^. By the time when all control animals had shown VO, only 30% of sh2612 animals had done so (Fig. 5c). Likewise, none of the sh2612 rats had ovulated at the age when all control rats had already completed this pubertal phase (Fig. 5d). In addition, estrous cyclicity was compromised (Fig. 5e, f), with the sh2612-injected animals showing fewer days in proestrus and many more days in an intermediate stage (estrous/diestrus) than C-injected rats (Fig. 5e, f).

To determine if the loss of *Mll1* targeted to the ARC resulted in the expected changes in gene expression, we injected another group of EJ rats and collected the MBH at LJ for mRNA quantitation. In agreement with the in vitro results, animals injected with sh2612 showed a pronounced reduction in *Mll1*, *Kiss1*, and *Tac3* mRNA abundance without alteration in the expression of *Pdyn* (Fig. 5g), the third member of the triad that, expressed in KDNy neurons, controls pubertal development. Importantly, sh2612 did not alter the expression of a number of other genes contributing to either repressing the pubertal process (*Eed*, *Mkrn3*, and *pEnk*; Fig. 5h) or involved in the stimulatory control of puberty (*Ttf1*, *Nell2*, *Eap1*, *Gpr54*, encoding the kisspeptin receptor; *Tac3R*, a preferred NKB receptor; *Grin2a* and *Grin2d*, encoding NMDA-type of glutamatergic receptors; and *Grik5*, encoding a kainate-type of glutamatergic receptor; Fig. 5i). The lack of a compensatory increase in *Eed* expression after loss of *Mll1* expression (Fig. 5h) is in agreement with a previous report showing that siRNA-induced *Mll1* knockdown in a different cell system does not result in increased PcG gene expression^26^. Thus, prepubertal loss of *Mll1* in the ARC selectively reduces *Kiss1* and *Tac3* expression, compromises the initiation and completion of puberty, and disrupts post-pubertal reproductive cyclicity.

### A *Kiss1* enhancer is activated before puberty

**Fig. 6:**
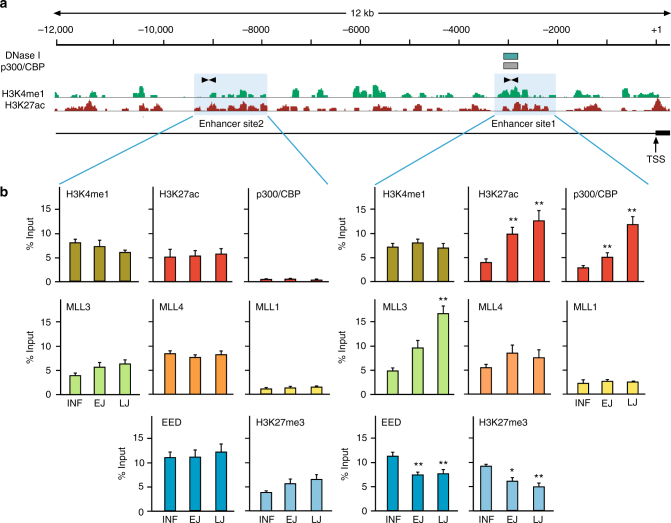
A genomic region upstream of the *Kiss1* gene behaves as a puberty-activated enhancer domain. **a** ChIP-seq tracts of H3K4me1 and H4K27ac within 12 kb of *Kiss1* 5′-flanking region detected using ARC chromatin from LJ female rats. TSS = transcription start site (ENSRNOT00000077054.1). ENCODE regions of DNase I hypersensitivity and p300/CBP binding from mouse forebrain are also shown (green and gray boxes, respectively). Two genomic regions (site 1 and site 2, shaded blue) enriched for H3K4me1 and H3K27ac were identified as putative enhancers of the *Kiss1* gene. **b** Abundance of H3K4me1 (dark green), H3K27ac (red), p300/CPB (red), MLL3 (light green), MLL4 (orange), MLL1 (yellow), EED (dark blue), and H3K27me3 (light blue) at enhancer sites 1 and 2 in the ARC throughout prepubertal development. Bars represent mean ±s.e.m. (*n* = 6–8 per group). *P < 0.05 and **P < 0.01 vs. INF; one-way ANOVA followed by SNK test

*Kiss1* expression has been postulated to be controlled by two different enhancers, one ARC-specific located upstream from the transcription start site (TSS)^27^, and one AVPV-specific located downstream from the 3′-end of the gene^28^. Because enhancers of active genes display high levels of H3K4me1 and H3K27ac^29^, we performed a genome-wide analysis of these two histone modifications in the MBH of LJ rats to identify potential enhancers of *Kiss1* transcription. A 12 kb segment of the *Kiss1* 5′ flanking region displayed two areas showing elevated levels of H3K4me1 and H3K27ac, one located between nucleotides (nt) −3188 and −2086 (site 1) and a more distal region (site 2) located approximately between nt −9400 and −7900 (Fig. 6a). Of these, site 1 is DNase I-hypersensitive in both mice and humans (Fig. 6a) and displays a histone mark signature typical of enhancers, including the presence of p300/CBP-binding sites (Fig. 6a; https://www.encodeproject.org/). Deletion of a genomic fragment containing site 1 results in selective loss of *Kiss1* expression in KNDy neurons, but not AVPV kisspeptin neurons of mice, indicating that site 1 is an ARC-specific *Kiss1* enhancer^27^. Consistent with this notion, analysis of site 1 and site 2 in the rat genome by targeted ChIP-qPCR revealed that only site 1 displayed significant association of p300/CBP, a hallmark of active enhancers^29,30^ and that the levels of both p300/CBP and H3K27ac increase significantly during prepubertal development (Fig. 6b). This demonstrates that site 1 is an enhancer that becomes active as the animal approaches puberty, and suggest that the previously postulated ARC-specific *Kiss1* enhancer^27^ resides in this genomic region. The COMPASS-like proteins MLL3 and/or MLL4 are responsible for depositing the H3K4me1 mark at distal enhancers and are required for p300/CBP-dependent H3K27 acetylation at these regions^19,20^. In keeping with this concept, MLL3 recruitment to site 1 (but not site 2) increased significantly between EJ and LJ (Fig. 6b). Noteworthy, the changes in MLL3 recruitment to site 1 appear to be specific to MLL3, as they were not accompanied by similar changes in MLL4 occupancy. Because p300/CBP is recruited to enhancer regions via physical association with MLL3 or MLL4, and requires this association to promote H3K27 acetylation^20^, these results suggest that the changes in p300/CBP observed at site 1 during juvenile development are MLL3-dependent. TrxG-p300/CBP-mediated activation of distal enhancers counteracts PcG-mediated silencing^20,31^ to facilitate the transition from a poised to an active configuration^31^.

Poised enhancers contain an abundance of H3K4me1, low levels of the activating histone mark H3K27ac, and high levels of the PcG-dependent repressive mark H3K27me3. Active enhancers also contain H3K4me1, but are enriched in H3K27ac and depleted of H3K27me3^29,32^. Our results show a loss of EED and H3K27me3 from site 1, but not from site 2, during juvenile development (Fig. 6b), suggesting that site 1 is an enhancer whose activity increases antedating the initiation of female puberty due to increased TrxG stimulation coupled to loss of PcG repression.

### Epigenome silencing of the ARC *Kiss1* enhancer

To determine if site 1 is indeed a biologically relevant ARC-specific *Kiss1* enhancer we subjected site 1 to epigenome editing using clustered regularly interspaced short palindromic repeats (CRISPR)-Cas9 technology and a catalytically inactive Cas9 (dCas9) from *Staphylococcus aureus* (SaCas9) fused to the Krüppel-associated box (KRAB) silencing domain from Kox1/ZNF10^33,34^. Because dCas9 binds, but does not cleave DNA, dCas9 functions as a DNA-binding domain that is targeted to genomic regions of interest by single-guide RNAs (sgRNAs) complementary to DNA sequences adjacent to a motif known as the protospacer adjacent motif (PAM)^35^. Since we employed dSaCas9, our sgRNAs were 22 nt long and targeted DNA sequences adjacent to the PAM motif NNGRRT/NNGRR^36^. When localized to genomic DNA by this targeting platform, the KRAB repressive domain facilitates the formation of a heterochromatin complex that includes the methyltransferase SETDB1 and the deacetylase HDAC1^37^.

**Fig. 7:**
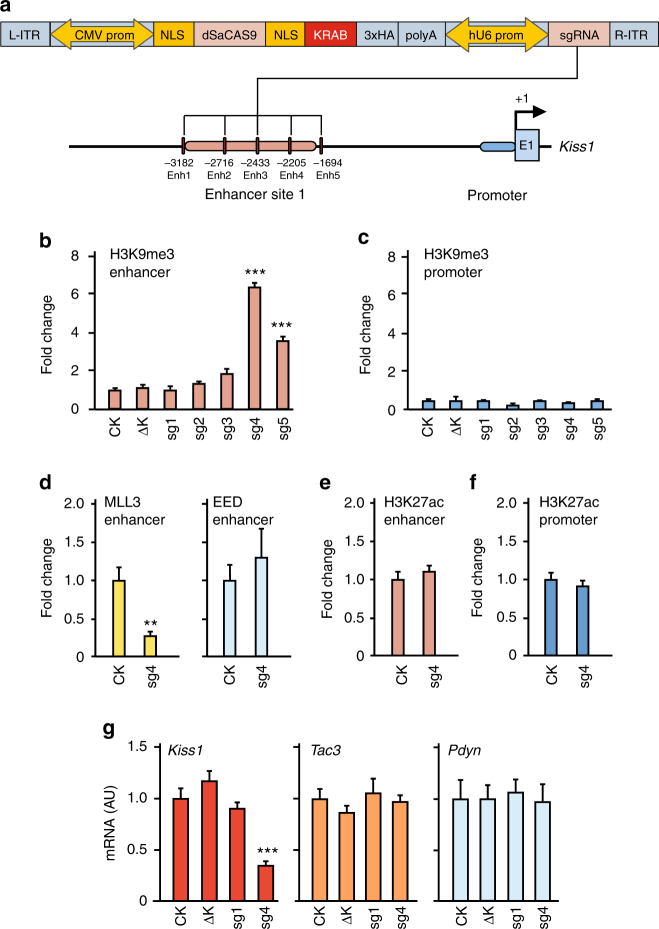
CRISPR-dSaCas9-KRAB-mediated epigenome silencing of the ARC *Kiss1* enhancer. **a** Schematic representation of the AAV-dSaCas9-KRAB construct used for epigenome editing of the *Kiss1* enhancer. The AAV plasmid backbone contains a CMV promoter driving expression of dSaCas9-KRAB, two nuclear localization signals (NLS) flanking dSaCas9, and three copies of an influenza hemagglutinin (3xHA) tag. It also contains a single-guide (sg) RNA cloning site and a U6 promoter driving sgRNA expression. sgRNAs complementary to five different sequences in site 1, the putative *Kiss1* enhancer domain, were identified using an online tool (https://www.deskgen.com/) and cloned into the AAV-dSaCas9-KRAB vector as recommended^66^. **b** H3K9me3 content at the *Kiss1* enhancer (site 1) or **c** Kiss1 promoter as assessed by ChIP-qPCR 2 days after transfecting Rat1 cells with AAV-dSaCas9-KRAB-sgRNAs 1–5 (sg1–5) or either of two control constructs, AAV-dSaCas9-KRAB (CK) or AAV-dSaCas9 (ΔK) (*n* = 3, experiment run in duplicate). **d** MLL3 and EED occupancy of the *Kiss1* enhancer site 1 in cells transfected with CK or dSaCas9-KRAB-sgRNA4 (sg4) (*n* = 3, experiment run in duplicate). **e**, **f** H3K27ac content at the *Kiss1* enhancer site 1 (**e**) and *Kiss1* promoter (**f**) in CK and sg4 transfected cells (*n* = 3, experiment run in duplicate). **g** *Kiss1*, *Tac3*, and *Pdyn* mRNA expression in Rat1 cells transfected with either CK (*n* = 6), ΔK (*n* = 4), sg1 (*n* = 6), or sg4 (*n* = 8). RNA expression data were normalized by using peptidylprolyl isomerase A as the housekeeping gene and dividing each individual value by the average of the CK group. ChIP-PCR data were also normalized by dividing each individual value by the average of the CK group. Bars represent mean ±s.e.m. (in **b**, **c**, **g**: ***P < 0.001 vs. control (CK) cells; one-way ANOVA followed by the Dunnett’s test; in **d**–**f**: **P < 0.01 vs. control (CK) cells; Student’s *t*-test). AU = arbitrary units

We prepared adeno-associated virus (AAV)-dSaCas9-KRAB-sgRNA constructs carrying five different sgRNAs targeting site 1 (Fig. 7a) and transfected these constructs into Rat1 cells. Two days later, we measured H3K9me3 (the histone mark catalyzed by SETDB1) and H3K27ac at site 1 by ChIP assays. Of the five constructs tested, the two sgRNAs targeting the 3′-end of site 1 (sg4 and sg5) were more effective in increasing H3K9me3 content (Fig. 7b). In contrast, none of the sgRNAs tested affected the abundance of H3K9me3 at the *Kiss1* promoter (Fig. 7c), indicating that sg4 and sg5 specifically established a repressive chromatin configuration at the *Kiss1* enhancer, leaving the chromatin landscape of the promoter unchanged. The control constructs dSaCas9-KRAB (CK) and dSaCas9-sg4 lacking the KRAB domain (ΔK), and the construct dSaCas9-KRAB-sg1 were similarly ineffective in altering H3K9me3 abundance (Fig. 7b), indicating that the effect of dSaCas9-KRAB-sg4 on H3K9me3 abundance at site 1 is specific and unrelated to a potential nonspecific effect of KRAB. The increase in H3K9me3 abundance at site 1 was accompanied by decreased MLL3 occupancy, without a significant change in EED recruitment (Fig. 7d), suggesting that KRAB-sgRNA-mediated silencing diminishes MLL3 access to the enhancer, without facilitating PcG recruitment.

In contrast to H3K9me3, basal levels of H3K27ac were not affected at either site 1 or the *Kiss1* promoter (Fig. 7e, f). This result is consistent with an earlier report showing that dCas9-KRAB does not alter H3K27ac content at another enhancer site^34^. Considered together, these two observations suggest that a change in basal H3K27ac content is not a core feature of KRAB-mediated epigenomic silencing. Instead, H3K9 methylation appears to be the hallmark of KRAB-mediated gene repression^37^, and the main feature of dCas9-KRAB-induced epigenetic silencing at enhancer sites^33,35^. In keeping with this view, *Kiss1* mRNA levels were markedly reduced by sg4, without alterations in either *Tac3* or *Pdyn* content (Fig. 7g). As was the case of H3K9me3 (Fig. 7b), AAV-dSaCas9-KRAB-sg1 (sg1) had no effect on *Kiss1* mRNA levels, which were similar to levels detected in cells exposed to either CK or ΔK (Fig. 7g). By demonstrating that epigenetic silencing of site 1 results in selective loss of *Kiss1* expression, these results identify site 1 as a bona fide ARC *Kiss1* enhancer.

### The ARC *Kiss1* enhancer is required for pubertal timing

**Fig. 8:**
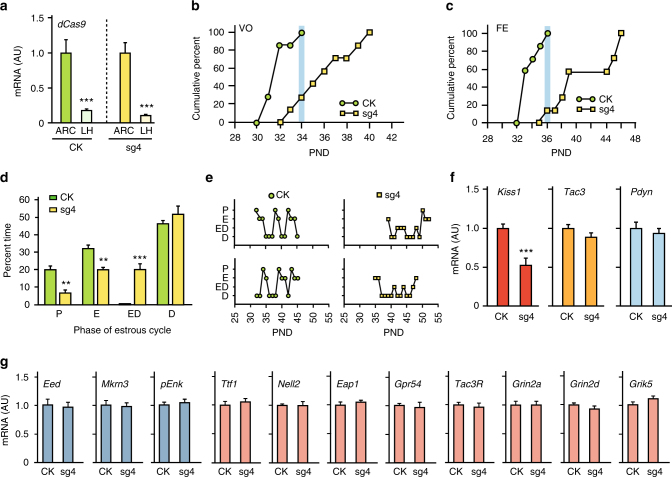
KRAB-sgRNA-mediated epigenome editing of the ARC *Kiss1* enhancer in the ARC of immature female rats.** a** dSa*Cas9* mRNA content in the ARC as compared with the lateral hypothalamus (LH) in rats microinjected bilaterally with sg4 or CK targeting the ARC. **b** Cumulative percent of animals showing vaginal opening (VO), and **c** first estrus (FE) in control CK or sg4-injected rats. Blue shade represents the time when all C animals had VO or FE. **d** Percent of time that rats injected with CK or sg4 spent in different stages of the estrous cycle. **e** Examples of estrous cycle patterns in rats injected with CK or sg4. **f** *Kiss1*, *Tac3*, and *Pdyn* mRNA levels in the ARC of animals injected with CK or sg4 detected by qPCR. **g** Expression of puberty-inhibitory genes (blue bars) or puberty-activating genes (magenta bars) in the ARC of female rats receiving CK or sg4. RNA expression data were normalized by using peptidylprolyl isomerase A as the housekeeping gene and dividing each individual value by the average of the CK group. P = proestrous; E = estrous; ED = transitional phase (estrous/diestrous); D = diestrous; AU = arbitrary units (*n* = 7–8 per group). Bars represent mean ±s.e.m. (**P < 0.01, ***P < 0.001 vs. CK-injected controls, Student’s *t*-test)

To determine the physiological consequences of silencing the ARC *Kiss1* enhancer in vivo we bilaterally targeted the ARC of EJ female rats with an AAV-dSaCas9-KRAB construct carrying sgRNA4 (sg4). Control (CK) animals were injected with the AAV-dSaCas9-KRAB construct devoid of sgRNAs (AAV-dSaCas9-KRAB, CK). We then followed the reproductive development of these animals to assess potential alterations in the time of puberty and reproductive cyclicity. Rats with correctly placed sg4 (determined by the elevated levels of *Cas9* mRNA in the ARC and a considerable lower expression in the lateral hypothalamus (Fig. 8a) had a markedly delayed age at VO (Fig. 8b; age at VO; C, 32.0 ± 0.35 days vs. sg4, 36.3 ± 0.90 days; *t* = 4.12, *P* < 0.01, Student’s *t*-test), and an even more pronounced delayed age at first estrous (Fig. 8c, age at first estrous; C, 33.9 ± 0.43 days vs. sg4, 41.3 ± 1.48 days; *t* = 4.46, *P* < 0.001, Student’s *t*-test). Because ovulation occurs on the day of the first estrous^9^, this result indicates that silencing the ARC *Kiss1* enhancer severely postpones not only the initiation but also the completion of puberty. Whereas all control animals had VO by PND34, only 29% of sg4 rats exhibited VO at this time (Fig. 8b). Similarly, only 14% of the sg4-injected rats had ovulated at the age when all control rats had already ovulated (Fig. 8c). In addition to these alterations, estrous cyclicity was strikingly disrupted, with the sg4-injected animals showing many more days in an intermediate stage (estrous/diestrus, ED) than in proestrus or estrous than controls (Fig. 8d, e).

To determine if silencing the ARC *Kiss1* enhancer in vivo resulted in a similar loss of *Kiss1* expression in the ARC as seen in cells in vitro, we collected the ARC of all animals at the diestrous phase and after all controls had undergone three complete estrous cycles, and measured *Kiss1*, *Tac3*, and *Pdyn* mRNA content, in addition to several other mRNAs (see below). In agreement with the in vitro results, *Kiss1* mRNA abundance was reduced in animals injected with sg4 as compared to controls (Fig. 8f). In contrast, neither *Tac3* nor *Pdyn* mRNA levels) were affected (Fig. 8f), as was the case for 11 other genes involved in the inhibitory (*Eed*, *Mkrn3*, and *pEnk*) and stimulatory (*Ttf1*, *Nell2*, *Eap1*, *Gpr54*, *Tac3R*, *Grin 2a*, *Grin2d*, and *Grik5*) control of puberty (Fig. 8g). Thus, in vivo epigenome silencing of the ARC *Kiss1* enhancer results in selective loss of *Kiss1* expression in the ARC, delays the initiation and normal progression of the pubertal process, and significantly compromises the normalcy of reproductive cyclicity.

## Discussion

Our results suggest that the timely initiation of female puberty requires a dual, coordinated action of COMPASS-like proteins exerted on *Kiss1 and Tac3*, two genes that—expressed in KNDy neurons of the hypothalamus—are essential for both puberty to occur and reproductive function to proceed^6,7,38^. On the one hand, MLL1 recruited to the promoter region of these two genes establishes a permissive chromatin configuration required for transcriptional activation. On the other hand, MLL3 acts at an ARC-specific *Kiss1* enhancer site to implement a chromatin structure that facilitates enhancer activation. As these changes take place, eviction of EED from the *Kiss1* enhancer and from the *Kiss1* and *Tac3* promoters results in loss of PcG-mediated repression^9^ at each of these genomic sites.

The earlier increase in H3K4me2 than in H3K4me3 abundance observed at both the *Kiss1* and *Tac3* promoters before puberty appears to be particularly relevant to the pubertal activation of *Kiss1* and *Tac3* expression. Methylation of lysine 4 at H3 follows an orderly sequence with monomethylation taking place first, followed by di- and trimethylation thereafter^20^. Additionally, trimethylation requires a longer interaction of the methylating enzyme with its unmethylated H3K substrate^14^. It is, therefore, likely that the earlier increase in H3K4m2 abundance at the *Kiss1* and *Tac3* promoter simply reflect the developmental unfolding of this biosynthetic sequence. It also implies that H3K4me2 facilitates the initial prepubertal activation of *Kiss1* and *Tac3* expression, which is then further enhanced and sustained by H3K4me3. Indeed, H3K4me2 is abundant at regulatory regions (such as promoters) targeted by transcription factors^39^, and at poised (i.e., ready to be activated) promoters^40^, such as *Kiss1* and *Tac3* before puberty. H3K4me3 abundance, on the other hand, is higher at the promoter of active genes^40^. Our results are consistent with these concepts as they show elevated levels of H3K4me2 at both the *Kiss1* and *Tac3* promoters preceding (*Kiss1*) or accompanying (*Tac3*) the prepubertal increase in expression of these genes, whereas H3K4me3 levels become elevated at the juvenile-pubertal transition (LJ period), coinciding with enhanced *Kiss1* and *Tac3* expression.

The critical importance that COMPASS-like proteins have for the coordinated regulation of these two puberty-activating genes, and hence for the pubertal process itself, is demonstrated by the striking delay of puberty and loss of reproductive cyclicity resulting from either ARC-specific RNAi-mediated knockdown of *Mll1* expression or epigenome silencing of the MLL3-supported ARC *Kiss1* enhancer. While RNAi-mediated loss of hypothalamic *Mll1* expression led to a reduction in both *Kiss1* and *Tac3* expression, CRISPR-dCas9-KRAB-sgRNA4-mediated epigenome silencing of the ARC *Kiss1* enhancer, resulted in selective loss of *Kiss1* expression. Yet, in both cases the net in vivo outcome was similar, as the advent and completion of puberty were delayed and reproductive cyclicity was disrupted. This similarity would be expected because *Kiss1* and *Tac3* operate along a common biological pathway in KNDy neurons.

The estrous cycle of both LV-sh2612 and AAV-dSaCas9-KRAB-sg4 (sg4)-injected animals showed a predominance of an intermediate stage between estrous and diestrus, suggesting that the basic recurrent pattern of pulsatile and surge LH release necessary for normal reproductive cyclicity was impaired. This long-term alteration of estrous cyclicity may be due to maintained loss of MLL1 in the former case, and persistent inhibition of enhancer activity by continuous replenishment of the heterochromatin complex drawn by the KRAB domain^37^ in the latter. Although epigenome silencing of the ARC *Kiss1* enhancer appeared to be more effective than ARC loss of *Mll1* in both delaying puberty and establishing a deranged pattern of estrous cyclicity, *Kiss1* mRNA levels in the ARC were similar in both groups implying that the seemingly different biological outcome of these manipulations may just reflect physiological variability in the response of animals treated in different experiments.

We demonstrate that CRISPR-dCas9-KRAB-mediated epigenome silencing of a distal enhancer alters a specific developmental milestone, such as puberty, in an in vivo setting. In agreement with previous in vitro results^33^, our findings also show that sg4-mediated epigenome editing increases the abundance of the repressive histone mark H3K9me3 only at the enhancer site, without affecting the content of this mark at the *Kiss1* promoter. In contrast to H3K9me3, the abundance of H3K27ac at the *Kiss1* enhancer remained unaltered, a result consistent with an earlier finding showing that dCas9-KRAB does not reduce the H3K27ac content of another enhancer^34^. While loss of H3K27ac may not be a core feature of KRAB-mediated epigenomic silencing, H3K9 methylation has been shown to be a hallmark of KRAB-mediated gene repression^37^, and the main feature of dCas9-KRAB-induced epigenetic silencing at enhancer sites^33,35^. Because the increase in H3K9me3 abundance at site 1 was accompanied by loss of MLL3 occupancy without increased EED recruitment, it can be concluded that exclusion of MLL3 from the *Kiss1* enhancer contributes to KRAB-mediated epigenome silencing, but that the repressive complex recruited by the KRAB-sgRNA platform does not involve increased EED occupancy. The above described changes were accompanied by decreased *Kiss1* expression in the absence of significant alterations in the expression of a host of other genes involved in the stimulatory and inhibitory control of puberty, indicating that dCas9-KRAB-sgRNA-mediated epigenome silencing targeting the *Kiss1* enhancer is a site-specific phenomenon. Consequently, our results provide a significant step forward toward defining the functional impact that modification of an enhancer has on a physiological function at an organismal level, a sought-after goal in genomic biology^29,41^. At a developmental neuroendocrine level, our results indicate that the timely activation of the ARC *Kiss1* enhancer is critical for both setting in motion the pubertal process and guiding it to completion.

Another interesting outcome of this study is the specificity of the MLL1 stimulatory effects on the two main puberty-activating genes expressed in KNDy neurons. MLL1 was recruited to the promoter region of only *Kiss1* and *Tac3*, but not to the promoter of the restraining gene *Pdyn*. In vitro experiments showed that in addition to the *Kiss1* and *Tac3* promoters, MLL1 is able to trans-activate the promoters of three other puberty-activating genes (*Ttf1*, *Nll2*, and *Eap1*). However, a transcriptome analysis of these and 10 additional genes involved in the stimulatory and inhibitory control of puberty, demonstrated that in no instance expression of these genes was reduced in the ARC of LV-sh2612-injected animals. A plausible explanation for this apparent discrepancy was provided by ChIP assays showing that in contrast to the puberty-related increase in MLL1 association to the *Kiss1* and *Tac3* promoters, MLL1 recruitment to the *Ttf1* and *Nell2* promoters (selected as examples of puberty-activating genes) does not increase at puberty. TTF1 is a transcription factor involved in the stimulatory control of *Kiss1* transcription^23^ and NELL2 is a protein selectively expressed in glutamatergic neurons that also contribute to facilitating female puberty^24^. These results suggest that although MLL1 is able to stimulate the transcription of genes other than *Kiss1* and *Tac3* under defined in vitro conditions, this effect might not occur at puberty, because recruitment of MLL1 to the promoters of these genes does not increase at this time.

Based on these considerations it can be concluded that an important function of MLL1 and MLL3 in the prepubertal hypothalamus is to steer the epigenetic balance controlling reproductive development via *Kiss1* and *Tac3* away from a repressive, PcG-mediated mode toward a predominantly stimulatory TrxG-driven status. By implementing histone modifications associated with transcriptional activation at specific genomic loci involved in the stimulatory control of puberty, the TrxG complex provides the necessary trans-activational drive to puberty-activating genes at the time when the strength of PcG-mediated epigenetic silencing is waning. The potential relevance of TrxG/PcG-dependent epigenetic regulation in the control of human puberty is suggested by the finding that inactivating mutations of CHD7^42,43^, a TrxG-related chromatin-remodeling protein that antagonizes PcG action, causes hypothalamic hypogonadism.

Altogether, the present results demonstrate a role of a PcG/TrxG-counterbalancing mechanism in the process by which the neuroendocrine brain controls the initiation of puberty, thereby broadening significantly the scope of developmental events controlled by PcG and TrxG proteins. In addition to being required for specification of early developmental processes^12^, our results suggest that PcG/TrxG-dependent balance of gene expression is a core component of one of the most fateful events of postnatal mammalian development, the acquisition of reproductive competence.

## Methods

### Animals

We used Sprague Dawley female rats (Charles River Laboratories International, Inc. (Hollister, CA) at four different phases of postnatal development: INF (PND14); EJ (PND21); LJ (PND28); and PUB (PND29-35). The Oregon National Primate Research Center (ONPRC) Animal Care and Use Committee approved the use of these animals in accordance with established NIH guidelines. The animals were obtained from, randomly assigned to different experimental groups, and housed in a room with controlled photoperiod (12/12 h light/dark cycle) and temperature (23–25 °C), with ad libitum access to tap water and pelleted rat chow.

Hypothalamic tissue from female rhesus monkeys (*Macaca mulatta*) was obtained through the ONPRC Tissue Distribution Program. Animals were classified into different stages of pubertal development based on the age of the animals and the pubertal stages reported by Watanabe and Terasawa^44^.

### Evaluation of sexual maturation and estrous cyclicity

We measured changes in hypothalamic gene expression and chromatin composition at three phases of female rat prepubertal development, INF, EJ, and LJ. At PND21 animals are in the EJ phase^45^. The vagina is closed and the uterus weighs 60 mg or less, and is devoid of intrauterine fluid. At PND28, the rats are considered to be in the LJ phase; the vagina remains closed and the uterus lacks fluid. LJ animals display a diurnal change in pulsatile plasma LH levels, with the amplitude of LH pulses increasing in the afternoons^46^. In rats, as well as in humans and nonhuman primates, this change signals the increase in hypothalamic drive that initiates puberty^47^.

To determine the effect of intrahypothalamic injections of a LV construct (LV-shMll1-GFP) targeting a shRNA to *Mll1* mRNA (sh2612) or an AAV vector (AAV-dSaCas9-sgRNA4, sg4) targeting the ARC-specific *Kiss1* enhancer on the onset of puberty and subsequent estrous cyclicity. Starting 5 days after the injections, the animals were inspected every morning for VO. Thereafter, we performed daily vaginal lavages to assess the occurrence of the first estrous, a stage in which most cells in the lavage are cornified. Vaginal cornification followed by a predominance of leukocytes was considered as proof that ovulation had occurred. An abundance of leukocytes defines the diestrous phase of the estrous cycle and indicates that a functional corpus luteum was formed after ovulation. Accordingly, the age at first ovulation was considered to have occurred only when the cornified cells were replaced by at least 2 days of vaginal lavages displaying mostly leukocytes^9^. In all cases, estrous cyclicity was monitored for at least 14 days after VO, a period during which control animals had experience at least four estrous cycles.

### Induction of a preovulatory surge of estrogen

To induce a preovulatory surge of plasma estradiol levels that sets in motion the LP phase of puberty, we injected 26-day-old female rats intraperitoneally (i.p.) with 5 IU of pregnant mare serum gonadotropin (Sigma-Aldrich, St. Louis, MO, USA), as previously described^48^. The MBH from each animal was collected 48 h later for molecular analysis.

### Tissue collection

The MBH of female rats was dissected by four cuts, one rostral adjacent to the posterior border of the optic chiasm, one caudal cut along the anterior edge of the mammillary bodies, and two lateral cuts placed between the medial eminence and the hypothalamic sulci. The fragments obtained had a thickness of about 2 mm. This fragment includes the entire ARC. The lateral hypothalamus collected in one experiment consisted of tissue dissected by lateral cuts made along the hypothalamic sulci and each of the two cuts previously made half-way between the hypothalamic sulci and the median eminence. A fragment of the CTX was also collected. Upon dissection, the tissues were immediately frozen on dry ice and stored at −85 °C until RNA or chromatin extraction. The MBH of female monkeys was dissected as described earlier^25^.

### Stereotaxic delivery of viral particles

We used both LV and AAV viral constructs (for description of these constructs see corresponding sections below). We delivered LV particles carrying an shRNA directed against *Mll1* mRNA (termed sh2612) or a control lentivirus expressing GFP (C) to the ARC of 21-day-old EJ female rats. Other animals received an AAV construct encoding a nuclease-deficient Cas9 (dCas9) fused with a KRAB repressor domain that is targeted to the ARC *Kiss1* enhancer sequence by a sgRNA (sg4). Control animals were injected with an AAV devoid of sgRNAs (CK). The animals were anesthetized with a cocktail of ketamine (25 mg ml^−1^), xylazine (5 mg ml^−1^), and acepromazine (1 mg ml^−1^), administered i.p. (0.15 ml per 100 g of body weight). Thereafter, the rats were positioned on a stereotaxic instrument (David Kopf Instruments, Tujunga, CA) with the incisor bar set at +5 mm. A total volume of 1 μl containing control viruses (C or CK), sh2612, or sg4 was injected bilaterally into the ARC at the rate of 250 nl min^−1^, using a 10 μl Hamilton micro-syringe connected to a Stoelting Stereotaxic microinjector (Stoelting, Wood Dale, IL). The coordinates used were as follows: 0.3 mm lateral from midline; 0.2 mm anterior from bregma; and 9.6 mm vertical from the surface of the skull^25^. The surgical procedure lasted about 15 min. Following surgery, the animals were placed in a clean cage on a heating pad until returned to their home cages. Thereafter, they were treated for 3 days with an analgesic (Carprofen, 5 mg kg^−1^) and an antibiotic (Baytril 10 mg kg^−1^), administered subcutaneously.

### RNA extraction, reverse transcription, and qPCR

Total RNA was extracted from tissues (MBH and CTX) and cultured cells using the RNeasy mini kit (Qiagen, Valencia, CA) following the manufacturer’s instructions. RNA concentrations were determined by spectrophotometric trace (Nanodrop, ThermoScientific, Wilmington, DE). Total RNA (500 ng) was transcribed into cDNA in a volume of 20 μl using 4 U Omniscript reverse transcriptase (Qiagen). We measured the mRNAs of interest using the SYBR GreenER™ qPCR SuperMix system (Invitrogen, Carlsbad, CA), and primers (Supplementary Table 1) designed with help of either DNASTAR 14 software (PrimerSelect tool, Madison, WI) or the Primer-Blast program from NCBI. All PCR reactions were carried out using a QuantStudio 12K Real-Time PCR system in a 10 μl volume, with 1 μl of cDNA or a reference cDNA (for details see below), 4 μl of primers (each primer at 1 µM each), and 5 μl of SYBR GreenER™ qPCR SuperMix. The PCR reaction was performed using the following conditions: 95 °C for 5 min, followed by 40 cycles of 15 s at 95 °C and 60 s at 60 °C. Formation of a single SYBR Green-labeled PCR amplicon was verified by running a three-step melting curve analysis after each reaction (15 s at 95 °C, 1 min at 60 °C, increasing to 95 °C at 0.5 °C s^−1^, with detection every 0.5 s, and ending at 95 °C for 15 s).

Threshold cycles (CTs) for each PCR reaction were identified using by QuantStudio 12K Flex software. To construct relative standard curves, we used serial dilutions (1/2 to 1/500) of a pool of cDNAs generated by mixing equal amounts of cDNA from each sample. The CTs from each sample were referred to the relative standard curve to estimate the mRNA content per sample; the values obtained were normalized for procedural losses using glyceraldehyde-3-phosphate dehydrogenase (*GAPDH*) mRNA or peptidylprolyl isomerase A (*Ppia*) as the normalizing unit.

### Massively parallel RNA sequencing

Total RNA from the MBH of female rats at different stages of prepubertal development was subjected to RNA-seq. The RNA-seq procedure was carried out by the OHSU Massively Parallel Sequencing Shared Resource. RNA-seq libraries were prepared using the TruSeq Stranded protocol with ribosomal reduction (Illumina, San Diego, CA). Briefly, 600 ng of total RNA per sample were depleted of ribosomal RNA using RiboZero capture probes (Illumina). The purified RNA was then fragmented using divalent cations and heat, and the fragmented RNA was used as template for reverse transcription using random hexamer primers. The resulting cDNAs were enzymatically treated to blunt the ends, and a single “A” nucleotide was added to the 3′-ends to facilitate adaptor ligation. Standard six-base pair Illumina adaptors were ligated to the cDNAs and the resulting DNA was amplified by 12 rounds of PCR. All of the above procedures were carried out following the protocol provided by Illumina. Unincorporated material was removed using AMPure XP beads (BeckmanCoulter, Brea, CA). Libraries were profiled on a Bioanalyzer instrument (Agilent, Santa Clara, CA) to verify: (a) the distribution of DNA sizes in the library; and (b) the absence of adapter dimers. Library titers were determined using real-time PCR (Kapa Biosystems, Wilmington, MA) on a StepOnePlus Real Time System (ThermoFisher, Waltham, MA). Libraries were mixed to run four samples per lane on the HiSeq 2500 (Illumina). Sequencing was done using a single-read 100-cycle protocol. The resulting base call files (.bcl) were converted to standard fastq formatted sequence files using Bcl2Fastq (Illumina). Sequencing quality was assessed using FastQC (Babraham Bioinformatics, Cambridge, UK).

### RNA-seq data analysis

To determine the differential expression of genes during pubertal development we used the gene-level edgeR^49^ analysis package. We performed an initial trimming and adapter removal pass using Trimmomatic^50^. Reads that passed Trimmomatic processing were aligned to the rn6 build of the rat genome with Bowtie2/Tophat2^51,52^, and assigned to gene-level genomic features with the Rsubread featureCounts package based on the Ensembl 83 annotation set. Differential expression between time points was analyzed using the generalized linear modeling approaches implemented in edgeR. Batch effect terms were included in these models to correct for runs on different dates/flow cells. Lists of differentially expressed genes/transcripts were identified based on significance of pairwise comparison of time points to identify the genes most likely to be differentially expressed for later reverse transcription (RT)-qPCR confirmation.

### Functional promoter assays

To determine if MLL1 alters the transcription of several putative downstream genes (*KISS1*,* TAC3*, *Ttf1*, *PDYN*, *Eed*, *Eap1*, *PENK*, *Nell2*, and *MKRN3*) we used Neuro2A cells (N2A, American Type Culture Collection (ATCC), Manassas, VA) transfected with luciferase reporter constructs containing the TSS and adjacent sequence of these genes (Supplementary Table 2) PCR-cloned into the *Sma*I/*Sac*I sites of pGL2 (Promega, Madison, WI), in addition to the expression vectors *Mll1-*pcXN2 (kindly provided by Thomas Milne, University of Oxford, Headington, Oxford, UK), *Eed*-pcDNA3.1, or a combination of both. The cells were cultured at 37 °C in a humidified atmosphere containing 5% CO_2_. They were maintained in Dulbecco’s modified Eagles medium (DMEM) containing high glucose (4.5 g l^−1^; Sigma), supplemented with 10% fetal bovine serum (FBS; Invitrogen), l-glutamine (2.5 mM, Sigma), 100 U ml^−1^ penicillin, and 100 µg ml^−1^ streptomycin (Invitrogen). For the assays, the cells (400,000 cells per well) were seeded onto 24-well plates in DMEM containing 10% FBS. After 24 h, we transiently co-transfected the reporter constructs (using as a backbone the luciferase reporter plasmid pGL2) along with the pcXN2-Mll1 construct and Lipofectamine 2000 (Invitrogen) at a ratio of 1 µg DNA:2.5 µl Lipofectamine 2000 in Optimem (Invitrogen). After 5 h of transfection, the cells were returned to serum-containing DMEM medium; 48 h later, they were harvested and lysed in 150 μl lysis buffer from the Firefly Luciferase Glow Assay Kit (Pierce, Rockford, IL). Cell lysate were spun at 10,000×*g* for 10 min and 100 μl supernatant was assayed for luciferase activity using 70 μl Luciferase reagent. All assays were carried out in opaque 96-well plates, and a Spectramax M5 microplate reader (Molecular Devices, Sunnyvale, CA) was employed to measure light emission. To assess transfection efficiency we co-transfected the plasmid CMV-Sport-β-gal (Invitrogen) at 10 ng ml^−1^, and determined β-galactosidase activity using 10 μl cell lysate and 100 μl Tropix Galacto Light Plus (ABI) reagent. β-Galactosidase activity was also determined in opaque 96-well plates and light emission was measured as indicated above.

### ChIP assay

To assess the recruitment of TrxG proteins to specific gene promoters, and the association of different histone modifications to either these promoters or putative distal enhancer domains in vivo, we performed ChIP assays using chromatin extracted from the MBH of prepubertal female rats (PND14, 21, and 28). In some cases, chromatin was also extracted from the CTX. To assess the changes in histone modifications resulting from expression of dSaCas9-KRAB-sgRNA constructs, ChIP assays were performed using chromatin extracted from Rat1 cells transfected with these constructs. The ChIP procedure was described previously by us^9,23^, and was carried out with minimal modifications. Cells were harvested for ChIP 48 h after transfection. The cells and tissue fragments were washed once in ice-cold phosphate-buffered saline (PBS) containing a protease inhibitor cocktail (PI, 1 mM phenylmethylsulfonylfluoride, 7 μg ml^−1^ aprotinin, 0.7 μg ml^−1^ pepstatin A, and 0.5 μg ml^−1^ leupeptin), a phosphatase inhibitor cocktail (PhI, 1 mM β-glycerophosphate, 1 mM sodium pyrophosphate, and 1 mM sodium fluoride), and an HDAC inhibitor (20 mM sodium butyrate). Thereafter, cells and tissue fragments were crosslinked by exposing them to 1% formaldehyde for 10 min at room temperature. After two additional washing steps in PBS the samples were lysed with 200 µl SDS buffer (0.5% SDS, 50 mM Tris-HCl, and 10 mM EDTA) containing protease, phosphatase, and HDAC inhibitors, and sonicated for 45 s to yield chromatin fragments of ~500 base pairs (bp) using the microtip of a Fisher Scientific FB 705 sonicator. Size fragmentation was confirmed by agarose gel electrophoresis. The sonicated chromatin was clarified by centrifugation at 14 000 r.p.m. for 10 min at 4 °C, brought up to 1 ml in Chip Dilution Buffer (16.7 mM Tris-HCl, pH 8.1, 150 mM NaCl, 1.2 mM EDTA, 1.1% Triton X-100, and 0.01% SDS) containing the PI and PhI cocktails, and the HDAC inhibitor described above. The samples were then stored at −80 °C for subsequent immunoprecipitation. For this step, chromatin was pre-cleared with Protein A/G beads (Dynabeads, Invitrogen) for 1 h at 4 °C. Twenty-five to 50 μl aliquots of chromatin were then incubated with 2–5 μg of the antibodies described in Supplementary Table 3. The complexes were incubated with 25 μl of protein A or G beads solution (Dynabeads) at 4 °C overnight with mild agitation. The next day the Ibeads were washed first with 0.5 ml low-salt wash buffer (20 mM Tris-HCl, pH 8.1, 150 mM NaCl, 2 mM EDTA, 1% Triton X-100, and 0.1% SDS), followed by high-salt wash buffer (20 mM Tris-HCl, pH 8.1, 500 mM NaCl, 2 mM EDTA, 1% Triton X-100, and 0.1% SDS), LiCl buffer (10 mM Tris-HCl, pH 8.1, 250 M LiCl, 1% Nonidet P-40, 1% sodium deoxycholate, and 1 mM EDTA), and finally with TE buffer (10 mM Tris-HCl, pH 8.0, and 1 mM EDTA). Thereafter, the immunocomplexes were eluted with 100 μl of 0.1 M NaHCO_3_ and 1% SDS at 65 °C for 45 min. To reverse the crosslinking reaction we added 4 μl of 5 M NaCl and incubated the samples at 95 °C for 30 min. We recovered the DNA using ChIP DNA Clean & Concentrator columns (Zymo Research, Irvine, CA), and stored the resulting material at −80 °C before qPCR analysis. All the chemicals mentioned above were purchased from Sigma-Aldrich.

### qPCR detection of chromatin immunoprecipitated DNA

Genomic regions of interest were amplified by qPCR. Accession numbers of the genes analyzed as well as the chromosomal position of the 5′-flanking region amplified, using the position of the TSS as the reference point, are shown in Supplementary Table 1. The primer sequences (Eurofins MWG Operon, Huntsville, AL) used to detect the DNA fragment of interest in the immunoprecipitated DNA are also shown in Supplementary Table 1. PCR reactions were performed using 1 μl of each immunoprecipitate (IP) or input samples (see below), primer mix (1 µM each primer), and SYBR Green Power Up Master Mix™ (Thermo Fisher) in a final volume of 10 μl. Input samples consisted of 10% of the chromatin volume used for immunoprecipitation. The thermocycling conditions used were as follows: 95 °C for 5 min, followed by 40 cycles of 15 s at 95 °C and 60 s at 60 °C. Data are expressed as % of IP signal/input signal.

### Genome-wide ChIP assays

To identify putative enhancer sites in the rat genome, ChIP assays were performed to assess the genome-wide distribution of H3K27Ac and H3K4me1 using chromatin extracted from the MBH of 28-day-old LJ female rats. The immunoprecipitated DNA was amplified before library preparation and deep-sequencing. Shortly, 10 µl of immunoprecipitated DNA were dephosphorylated using Shrimp Alkaline Phosphatase (NEB, 1 U) for 10 min at 37 °C. Thereafter, thymidine residues were added to the DNA ends using Terminal Transferase (NEB, 20 U), in a T-tailing reaction (20 min at 37 °C) that utilized 5 μM dTTP and 5 μM ddCTP. Upon completion of this reaction, a oligodeoxynucleotide adapter [T7-BpmI-oligo(A)15 (5′-AATTAATACGACTCACTATAGGGCTGGAGAAAAAAAAAAAAAAA-3′; 0.2 μM; Operon) was annealed to the ends of the DNA fragments and extended using 0.2 mM dNTPs and Klenow fragment polymerase (NEB, 5 U) for 1 h at 37 °C. RNA was then synthesized by in vitro transcription using T7 RNA polymerase and the RNAMaxx high yield kit (Agilent) in an overnight reaction at 37 °C. The resulting RNA was purified using the RNeasy Mini Kit (Qiagen) and eluted in 20 µl sterile water. Complementary DNA was then prepared using the T7-BpmI-oligo(A)15 and the Superscript III reverse transcription Kit (Invitrogen), followed by second-strand synthesis using a 1:5 ratio TaqPolymerase (Roche, Branford, CT) and Pfu Polymerase (Agilent) in Thermopol Buffer (NEB) in a 200 µl reaction for 30 min at 72 °C. The double-stranded DNA was purified using the QiaQuick PCR purification Kit (Qiagen) and eluted in 50 µl sterile water. The purified DNA was then digested with *Bpm*I (NEB) in a 60 µl reaction for 2 h at 37 °C, followed by isolation using Zymo ChIP columns (Zymo Resaerch, Irvine, CA) and elution in 20 μl sterile water. Eluted DNA was quantitated using the Qubit High Sensitivity Kit (Invitrogen) and the DNA integrity was assessed using a Bioanalyzer High Sensitivity Chip (Agilent). The amplified DNA was then used for library preparation.

This procedure and massively parallel sequencing were performed by the Center for Genome Research and Biocomputing (Oregon State University). The DNA libraries were generated, PCR-amplified, and quantified as described above for RNA-seq. The resulting samples were run on a HiSeq 3000 with a targeted density of 300 million reads per lane, and using a single-read 100-cycle protocol. The data were analyzed as outlined above for RNA-seq.

### ChIP-seq data analysis

To study chromatin transitions by ChIP-seq analysis, sequences were filtered using Trimmomatic and mapped to the rn6 reference rat genome using the Bowtie2 aligner as described above (RNA-seq data analysis). To identify the regions of the genome enriched in H3K27ac and H3K4me1, we visualized the alignment of mapped reads using the Integrated Genome Viewer^53^. We also utilized MACS^54^ to identify individual peaks for each epigenomic mark within each sample. We utilized a control based on random shuffling of the original alignments rather than input DNA for MACS analysis as such controls have been shown to be nearly as effective as input DNA^55,56^.

### Fluorescent in situ hybridization

For this procedure we used four LJ 28-day-old female rats. Following intracardiac perfusion with 4% paraformaldehyde borate buffer, pH 9.5, we processed the brains for hybridization histochemistry, as described^57,58^. We used the double FISH procedure described by Watakabe et al.^59^ employing various complementary (c)RNA probes. Probes complementary to a COMPASS mRNA (*Set1b*) and COMPASS-like mRNAs (*Mll1/KMT2a*, *Mll2/Kmt2b*, *Mll3/Kmt2c*, and *Mll4/Kmt2d*) were labeled with fluorescein-12-UTP (FITC). A *Kiss1* cRNA probe was labeled with digoxigenin-11-UTP (Dig). The labeling reactions were performed in a 10 µl volume, containing 1 µl of a 2 mM digoxigenin-UTP solution; 500 ng of cDNA template; 2.5 mM of each ATP, CTP, and GTP; and 15–20 U of SP6 RNA polymerase. Following 1 h of incubation at 40 °C the reaction was treated with DNase, stabilized with dithiothreitol (DTT) and salt, and the volume was adjusted to 100 µl with 20 mM DTT. The cRNA probe was precipitated with ethanol, dried, and resolubilized in 100 µl diethyl pyrocarbonate-treated water. Five microliters of the mixture were then run on a formaldehyde agarose gel to assess the integrity of the cRNAs and the yield of the reaction, as reported^60^.

Control sections were incubated with sense probes transcribed from the same plasmid, but linearized on the 3′-end to transcribe the coding strand of the cDNA template. Following treatment with proteinase K and acetic anhydride, the sections were hybridized overnight at 55 °C with Dig-*Kiss1* cRNA in combination with either FITC-COMPASS mRNA (*Set1b*) or FITC-COMPASS-like mRNAs (*Mll1/KMT2a*, *Mll2/Kmt2b*, *Mll3/Kmt2c*, and *Mll4/Kmt2d*). The next day, the slides were washed at high stringency (final wash: 0.1× sodium chloride-sodium citrate at 65 °C for 30 min). Thereafter, the sections were incubated with 0.3% H_2_O_2_ for 10 min to block endogenous peroxidases, followed by 30 min in 0.5% blocking reagent provided with the Renaissance tyramine signal amplification (TSA) Plus dinitrophenyl (DNP) system (PerkinElmer, Boston, MA) used to enhanced the FITC reaction. Following these blocking steps, the sections were incubated overnight at 4 °C simultaneously with antidigoxygenin-alkaline phosphatase-conjugated sheep antibodies (Roche, Indianapolis, ID) diluted 1:1000 and a mouse monoclonal anti-FITC antibody conjugated to a peroxidase conjugated IgG fraction (Jackson ImmunoResearch Laboratories, West Grove, PA) diluted 1:4000 in TNT buffer (0.1 M Tris-HCl, 0.15 M NaCl, and 0.5% Triton X-100). The next day, the sections were washed in TNT buffer (three times, 10 min each) before a 30 min incubation at room temperature with TSA Plus DNP reagent (PerkinElmer) diluted 1:50. Following three washes in TNT buffer (10 min each), the sections were incubated with rabbit anti-DNP-keyhole limpet hemocynin Alexa 488 antibodies (Invitrogen/Molecular Probes, Eugene, OR) diluted in TNT buffer for 2 h at room temperature to develop the FITC reaction into green fluorescence. Thereafter, the sections were again washes in TNT buffer (three times, 10 min each time) followed by one wash in TS8.0 buffer (0.1 M Tris-HCl, pH 8.0, 0.1 M NaCl, and 10 mM MgCl_2_); the digoxigenin reaction was then converted to red fluorescence by incubating the sections with HNPP (2-hydroxy-3-naphtoic acid-2′-phenylanilide phosphate)/Fast Red reagent (Roche) for 30 min at room temperature. After three washes with in PBS-10 mM EDTA, the sections were incubated with Hoechst 33258 (Invitrogen) at 0.1 µg ml^−1^ for 1 min, washed in PBS, and coverslipped with aqueous mounting medium, before fluorescence microscopy examination^9^.

### Probes for in situ hybridization

We employed several cRNAs. A *Kiss1* cRNA probe was prepared by transcribing a 393 bp rat *Kiss1* cDNA^61^ (nt 1–393 in rat *Kiss1* mRNA; accession No. NM_181692.1). A Set1b cRNA probe was generated by transcribing a 535 bp cDNA template (nt 7390–7925 in the coding region of rat *Set1b* mRNA; accession No. XR_001836008.1). An *Mll1* cRNA was transcribed from a 522 bp cDNA template (nt 596–1118 in rat *Mll1* mRNA; accession No. XM_008766179.1). An *Mll2* cRNA was transcribed from a 438 bp cDNA fragment (nt 31–469 in rat *Mll2* mRNA; accession No. XM_017595318.1). An *Mll3* cRNA was transcribed from a 425 bp cDNA template (nt 5009–5434 in rat *Mll3* mRNA; accession No. XM_006235840.3). Finally, an *Mll4* cRNA was transcribed from a 630 bp cDNA fragment (nt 6753–7383 in rat *Mll4* mRNA; accession No. XM_008759254.2).

All cRNA probes were prepared by in vitro transcription of cDNA templates generated by RT-PCR amplification of hypothalamic total RNA. The PCR fragments were cloned into the pGEM-T vector (Promega) and their identity was verified by sequencing. Primer sequences and regions recognized by the cRNA probes used are shown in Supplementary Table 1.

### Small hairpin RNAs and lentivirus construct design

To identify shRNA sequences able to reduce *Mll1* mRNA expression, sense and antisense 22-mer oligodeoxynucleotides **(**Supplementary Table 1) encoding four different shRNAs were designed using the online tool https://sispotr.icts.uiowa.edu/sispotr/index.html;jsessionid=245170F2742B69FB408F737CCE6DAC8F. The sense and antisense sequences were incorporated in silico (http://cancan.cshl.edu/cgi-bin/Codex/Tools.cgi) into a pPRIME sequence to generate 97-mer oligodeoxynucleotides, which we then purchased from ThermoFisher as PAGE-purified reagents. To convert these long oligodeoxynucleotides into double-stranded DNA, we PCR-amplified them using pSM2C^62^ forward (5′-GATGGCTGCTCGAGAAGGTATATTGCTGTTGACAGTGAGCG-3′) and reverse (5′-GTCTAGAGTCTAGACGAGGCAGTAGGCA-3′) primers. The forward primer contains a *Xho*I restriction site at the end, and the reverse primer contains an *Eco*RI site (double underlined)^62^. For amplification we utilized 100 ng of the template DNA, 25 pmol of the forward and reverse synthesis primers, and 1 unit of VentR taq polymerase (New England Biolabs, Ipswich, MA). The PCR reaction consisted of 94 °C for 5 min, 12 cycles of 94 °C, 54 °C, and 75 °C (30 s each), and a final incubation at 75 °C for 2 min. The resulting PCR products were 132 bp in length. Following restriction enzyme digestion, the double-stranded DNA fragments, which generate the required hairpin structure^62^, were 110 bp. They were ligated into the *Xho*I–*Eco*RI sites of the pPRIME microRNA LV vector system, as recommended^62^. The pPRIME vectors contain a RNA polymerase II promoter that directs expression of a marker gene, which in our construct encodes an eGFP transcribed under the control of the cytomegalovirus (CMV) promoter (Supplementary Fig. 7a). To identify the cells transduced by the virus following either in vitro or in vivo infection we detected eGFP by immunohistofluorescence^25^. The eGFP-encoding sequence is located immediately upstream from an artificial miRNA precursor derived from miR-30^63^. This miRNA sequence contains adjacent *Xho*I and *Eco*RI sites (Supplementary Fig. 7a) that allow replacement of the hairpin sequence with shRNAs targeting the mRNA of interest^62,64^. A siRNA generated against firefly luciferase mRNA^62^, but containing nonsense mutations, was used as a control (C). Because of the mutations introduced, this siRNA targets neither mammalian mRNAs nor the firefly luciferase mRNA^62^. After confirming the sequence of each shRNA construct, infective LV particles were prepared by transient co-transfection of the vector plasmid and the packaging plasmids into 293T cells. The virus was concentrated by ultra-centrifugation, and the resulting pellet was resuspended in Hanks balanced salt solution. The packaging and preparation of the virus has been described in detail elsewhere^65^.

### Efficiency of LV-Mll1-GFP to reduce *Mll1* expression in vitro

The ability of the shRNA-Mll1 constructs to reduce *Mll1* mRNA levels was tested and compared to the control virus (C) using the immortalized R22 hypothalamic cell line (Cedarlane, Burlington, NC). The cells were plated in DMEM medium at 400,000 cells per well using 12-well plates. Twenty-four hours later, the cells were transduced with the viruses at a multiplicity of infection of 5–1. Three days after the infection, transduced cells (identified by their expression of eGFP) were isolated by flow cytometry to produce a pure population of cells. These cells were expanded and re-plated onto 12-well plates at a density of 300,000 cells per plate. Three days later, total RNA was extracted as described above and *Mll1* mRNA levels were determined by qPCR using the primers listed in Supplementary Table 1.

### Preparation of AAV-dSaCas9-KRB-sgRNA constructs

All plasmids used to generate a catalytically deactivated (d) *Staphylococcus aureus* (Sa)Cas9-KRB-sgRNA construct were purchased from Addgene (https://www.addgene.org/). We first excised a 3357 bp DNA fragment containing the coding region of dSaCas9 from plasmid pX603-AAV-CMV::NLS-dSaCas9 (D10A,N580A)-NLS-3xHA-bGHpA using *Age*I and *Eco*RI (ThermoFisher) restriction endonucleases. We then removed a DNA fragment containing the coding region of wild-type SaCas9 from pX601-AAV-CMV::NLS-SaCas9-NLS-3xHA-bGHpA;U6::BsaI-sgRNA also using *Age*I and *Eco*RI, and replaced this DNA segment with that encoding dSaCas9. We termed the new vector pX601-AAV-CMV::NLS-dSaCas9-NLS-3xHA-bGHpA;U6::BsaI-sgRNA. Next, we PCR-amplified from pHR-SFFFV-dCas9-BFP-KRAB a 222 bp DNA fragment containing the KRAB domain using In-fusion PCR technology (Takara, Mountain View, CA) and cloned this fragment into the *Bam*HI site that follows the nuclear localization signal (NLS) at the 3′-end of the dSaCas9 sequence in pX-AAV-CMV::NLS-dSaCas9-NLS-3xHA-bGHpA;U6::BsaI-sgRNA (Supplementary Fig. 6). The primers used are shown in Supplementary Table 1. The resulting vector is termed AAV-dSaCas9-KRAB-3xHA-U6::Bsa1-sgRNA. The integrity of each component added to the original pX601-AAV-CMV::NLS-SaCas9-NLS-3xHA-bGHpA;U6::BsaI-sgRNA was verified by sequencing. The construct (sg4) found to increase H3K9me3 deposition at the ARC *Kiss1* enhancer most efficiently was modified to generate a new control (ΔK) devoid of the KRAB domain. We used *Bam*HI and *Eco*RI to remove the KRAB encoding DNA, T4 DNA polymerase (New England Biolabs) to blunt the open ends, and T4 DNA ligase (New England Biolabs) for plasmid re-ligation.

### Preparation of sgRNAs

A panel of five sgRNAs targeting the putative *Kiss1* enhancer site 1 was designed and cloned. The sgRNAs were designed using an online tool (https://www.deskgen.com/) searching for the PAMs NNGRRT and NNGRR^36^. Following phosphorylation and annealing of each oligonucleotide set, the double-stranded products were digested with *Bsa*I (*Eco*31I), and ligated into the *Bsa*I site of AAV-dSaCas9-KRAB-3xHA-U6::Bsa1-sgRNA. After treatment with Plasmid Safe DNA exonuclease (Epicentre, Madison, WI), 2 µl of each reaction were used to transform Stbl3 cells (ThermoFisher). The next day, colonies were collected and grown overnight for DNA extraction and sequencing. The identity of all sgRNAs was confirmed by sequencing using a primer (Supplementary Table 1) complementary to the U6 promoter sequence^66^.

### Evaluation of AAV-dSaCas9-KRAB-sgRNA biological activity

To assess the biological activity of these sgRNAs we used Rat1 cells (ATCC CRL-2210). The cells were plated in the medium described under Functional promoter assays at 200,000 cell per well, using 12-well plates (for qPCR) or 400,000 cells per well in 6-well plates (for ChIP assays).

For chromatin extraction, the cells were transfected 24 h after plating with the AAV-dSaCas9-KRAB-3xHA-U6::Bsa1-sgRNA constructs using Lipofectamine 3000 (Invitrogen) at a ratio 1 µg DNA: 3 µl Lipofectamine 3000 in 100 μl Optimem (Invitrogen). Control cells were transfected with either CK or ΔK. 48 h after transfection, the cells were snap-frozen before measuring by ChIP-qPCR the content of H3K9me3 and H3K27ac at the *Kiss1* enhancer site 1 and *Kiss1* promoter.

For *Kiss1*, *Tac3*, and *Pdyn* mRNA measurements, we treated the cells with 5-azacytidine (1 nM; Sigma-Aldrich) to reduce DNA methylation that may be repressing gene expression^67^. The treatment was initiated 24 h before transfection and was maintained until the end of the experiment, i.e., 48 h after transfection. Because the AAV-dSaCas9-KRAB-3xHA-U6::Bsa1-sgRNA constructs we used do not carry a GFP marker, and thus do not allow fluorescent-based cell sorting, we co-transfected each construct (1 μg per 1 × 10^6^ cells) with pcDNA-GFP (0.2 µg per 1 × 10^6^ cells; 5:1 ratio). Forty-eight hours after co-transfection, the cells expressing GFP were isolated by FACS, and frozen at −80 °C until *Kiss1*, *Tac3*, and *Pdyn* mRNA levels were determined by qPCR.

### Detection of hypothalamic LV infection

Cells transduced with C or sh2612 were identified in 35 μm brain sections by immunohistofluorescence using a goat polyclonal antibody against eGFP (1:2000, AbCam, Cambridge, MA; Supplementary Table 3). Following an overnight incubation with this antibody, the sections were incubated for 1 h at room temperature with an Alexa 488 donkey antigoat IgG (1:500, Invitrogen), followed by 1 min incubation with Hoechst 33258 reagent (Thermo Fisher; 1:10,000) to stain cell nuclei.

### Detection of dCas9 mRNA in the hypothalamus

To assess the accuracy of the intrahypothalamic injections of sg4 we used qPCR to measure the levels of *dCas9* mRNA attained in the ARC as compared with the lateral hypothalamus at the time of completion of the study. The primers used are shown in Supplementary Table 1.

### Statistics

All statistical analyses were performed using Prism 7.0 software (GraphPad, San Diego, CA). The data were first subjected to a normality and an equal variance test. Data that passed these two tests were then analyzed by either analysis of variance (ANOVA) followed by the Student–Newman–Keuls to compare multiple groups, the Dunnett’s test to compare several groups to a single control group, or the Student’s *t*-test to compare two groups. When comparing percentages, groups were subjected to an arc–sine transformation before statistical analysis to convert the values from a binomial to a normal distribution^68^. The sample size was selected based on power analyses performed using the s.d.’s that we normally observe when measuring the parameters examined in this study and an *n* = 6 per group. These analyses provide at least 80% (type II error = 0.124) power to detect two effect sizes using either ANOVA or two-sided two-sample *t*-test with a significance level of 0.05. The investigator was blinded to the group allocation in all physiological and molecular determinations.

### Data availability

The RNA-seq and ChIP-seq data have been deposited into the Gene Expression Omnibus hosted at the National Center for Biotechnology Information with the accession numbers GSE94080 and GSE95660 respectively.

## Electronic supplementary material

Supplementary Information
