# Supplementary material for: Trithorax dependent changes in chromatin landscape at enhancer and promoter regions drive female puberty
Source: Nat Commun. 2018 Jan 4;9:57. doi: 10.1038/s41467-017-02512-1 (PMC5754362; doi:10.1038/s41467-017-02512-1)
Supplement: Supplementary file 1 — Supplementary Information [file 41467_2017_2512_MOESM1_ESM.pdf]

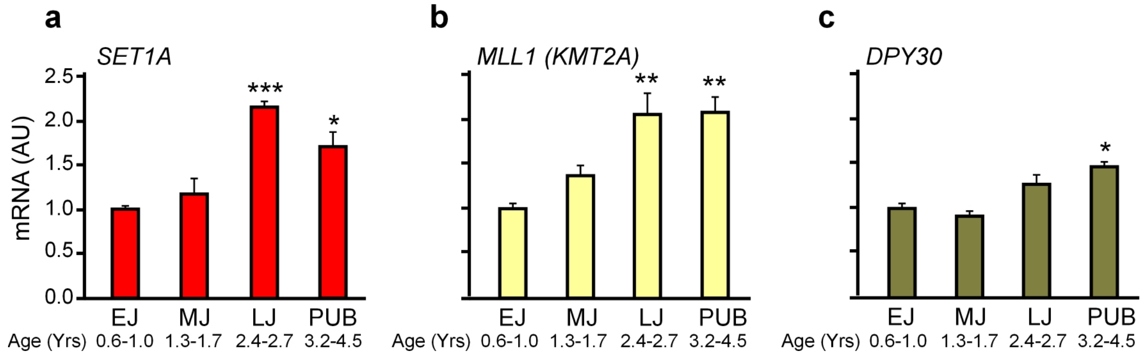

Supplementary Fig. 1: Expression of TrxG genes in the immature female rhesus monkey hypothalamus. (**a**) *SET1A* mRNA**;** (**b**) *MLL1 (KMT2A*) mRNA; (**c**) *DPY30* mRNA. RNA expression data were normalized by dividing each individual value by the average of the EJ group Bars represent mean ± s.e.m. (n=3-7) (* = P<0.05; ** = P<0.01, and *** = P<0.001 vs EJ group; one-way ANOVA-SNK test. EJ, early juvenile; MJ, midjuvenile; LJ, late juvenile; PUB, peripubertal.

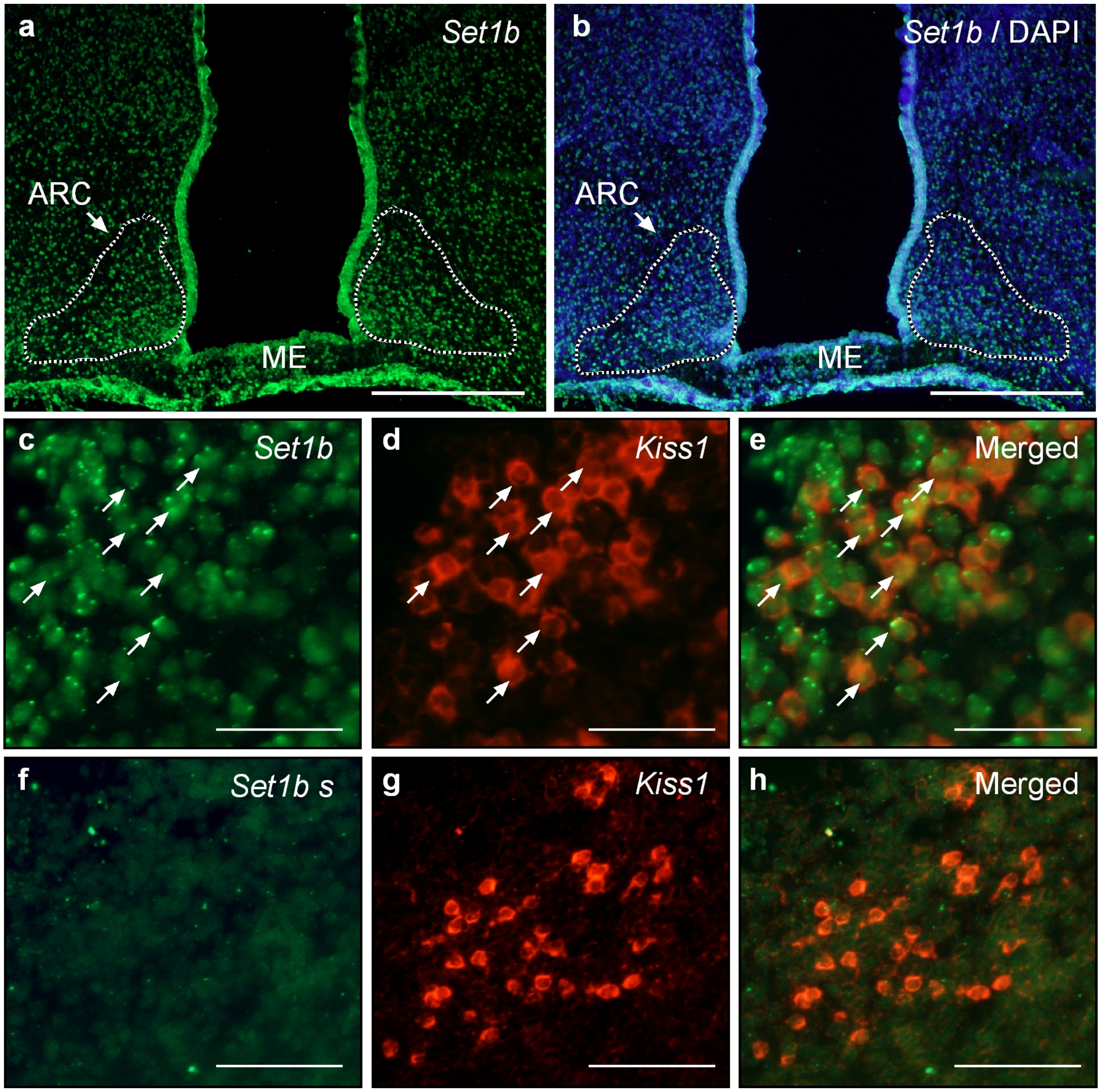

Supplementary Fig. 2: *Set1b* mRNA-expressing cells in the prepubertal female rat hypothalamus. (**a**) Low magnification view depicting the distribution of *Set1b* mRNA-containing cells in the MBH of LJ female rat as assessed by fluorescent *in situ* hybridization (FISH). The dotted line outlines the boundaries of the ARC. (**b**) Abundance of *Set1b* mRNA-containing cells (green) in the ARC as assessed by FISH using the DNA marker DAPI (blue) to identify cell nuclei. (**c-e**) Co-localization of *Set1b* mRNA (green) and *Kiss1* mRNA (red) in KNDy neurons of the ARC. Arrows point to examples of double positive cells. (**f-h**) Absence of *Set1b* mRNA hybridization in sections incubated with a sense (s) *Set1b* RNA probe (green) and a *Kiss1* cRNA (red). ME = median eminence of the hypothalamus. Bars in a, b = 500 μm; in c-h = 200 μm.

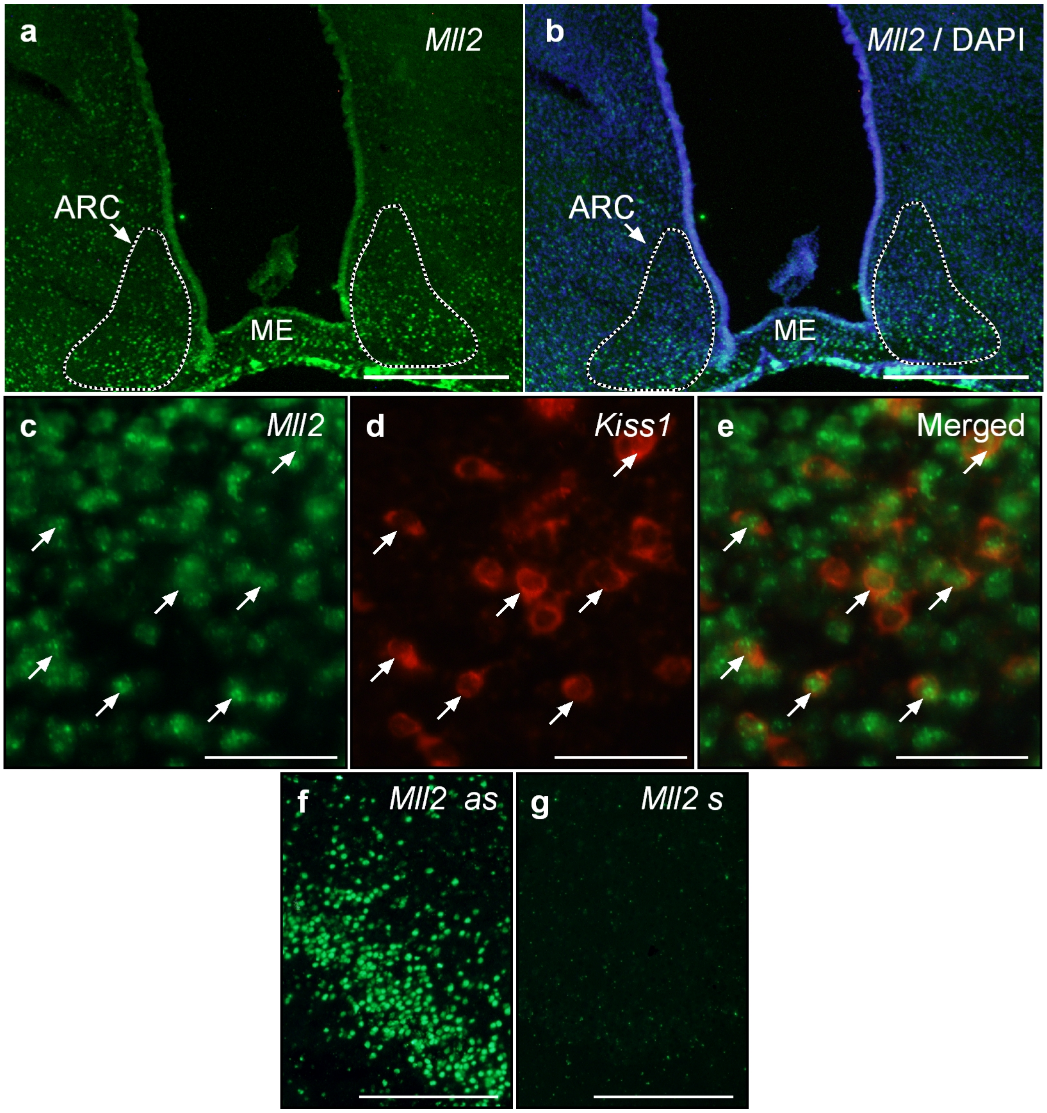

Supplementary Fig. 3: *Mll2* mRNA- expressing cells in the prepubertal female rat hypothalamus. **(a)** Low magnification view depicting the distribution of *Mll2* mRNA-containing cells in the MBH of a LJ female rat as assessed by FISH. The dotted line outlines the boundaries of the ARC. (**b**) Abundance of *Mll2* mRNA-containing cells (green) in the ARC as assessed by FISH using the DNA marker DAPI (blue) to identify cell nuclei. (**c-e**) Co-localization of *Mll2* mRNA (green) and *Kiss1* mRNA (red) in KNDy neurons of the ARC. Arrows point to examples of double positive cells. (**f, g**) Absence of *Mll2* mRNA hybridization in sections incubated with a sense (s) *Mll2* RNA probe (green) (g), as compared to an adjacent section incubated with an *Mll2* cRNA (f). Bars in A, B = 500 μm; in C-G = 200 μm.

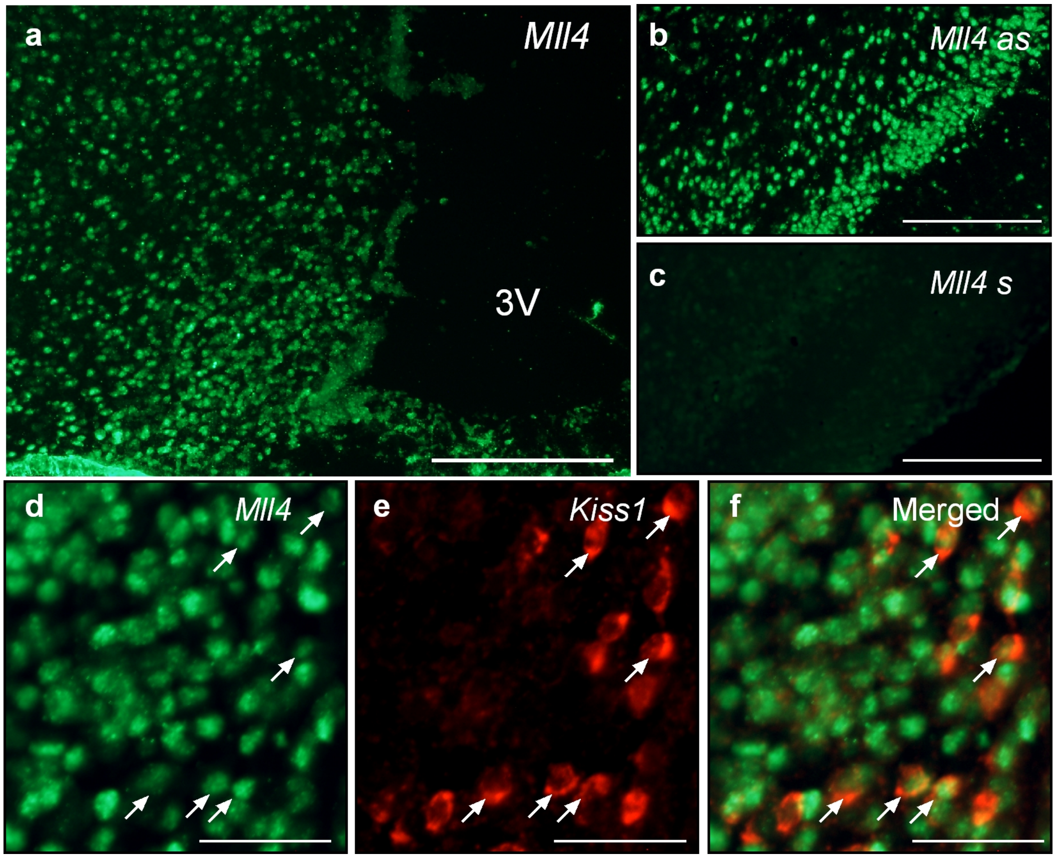

Supplementary Fig. 4: *Mll4* mRNA-expressing cells in the prepubertal female rat hypothalamus. **(a)** Low magnification view depicting the distribution of *Mll4* mRNA-containing cells in the MBH of a LJ female rat as assessed by FISH. (**b, c**) Absence of *Mll*4 mRNA hybridization in sections incubated with a sense (s) RNA probe (c), as compared to an adjacent section (b) incubated with an *Mll4* cRNA probe (green). (**d-f)** Co-localization of *Mll4* mRNA (d, f; green and *Kiss1* mRNA (e, f; red) in KNDy neurons of the ARC. Arrows point to examples of double positive cells. Bars in a = 500 μm; in b-f = 200 μm.

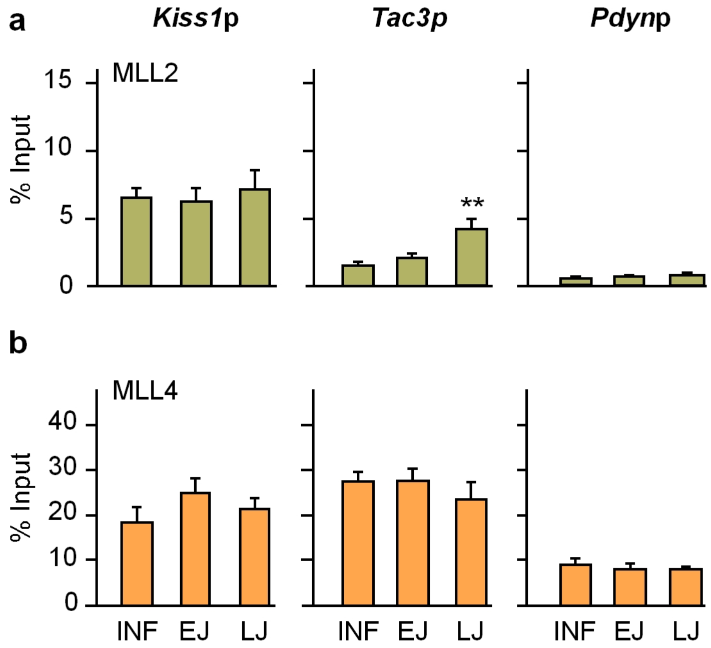

Supplementary Fig. 5: Recruitment of MLL2 and MLL4 to the promoter of KNDy genes. (**a, b**) Recruitment of MLL2 (a) and MLL4 (b) to the *Kiss1*, *Tac3* and *Pdyn* promoters in the MBH of LJ female rats. (n=7-8/group). Each bar represents the mean ± SEM. (** = P<0.01 vs. INF group, one-way ANOVA-SNK test.

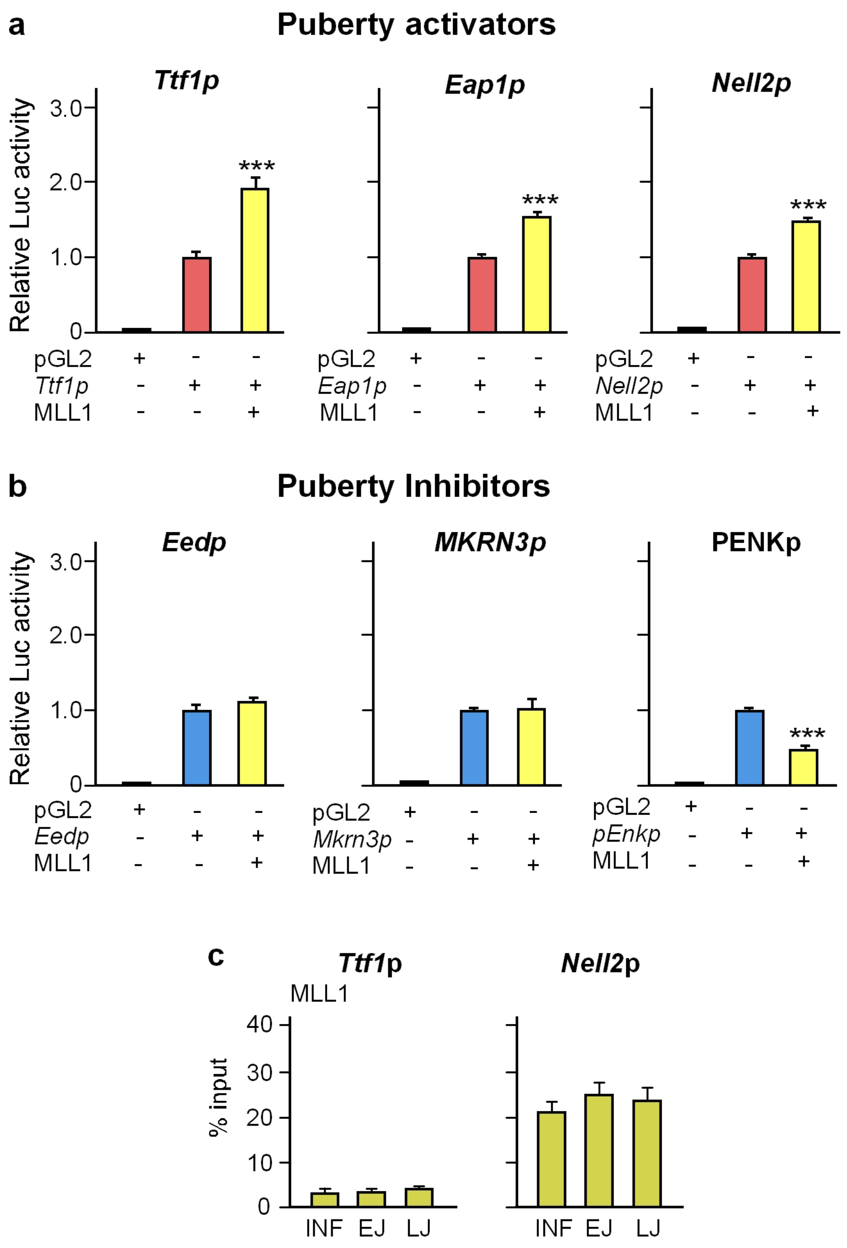

Supplementary Fig. 6: Effect of MLL1 on promoter activity and MLL1 binding to promoters during prepubertal development. (**a**) Effect of MLL1 on *Ttf1, Eap1* and *Nell2* promoter activity (n=6). (**b**) Effect of MLL1 on Eed, MKRN3 and PENK promoter activity (n=6). Each bar represents the mean ± s.e.m. *** = p<0.001 vs. cells transfected with luciferase constructs containing the indicated promoters; one-way ANOVA-Dunnett's test). (**c**) MLL1 recruitment to the *Ttf1* and *Nell2* promoters during prepubertal development (n=8). Each bar represents the mean ± s.e.m. *** = *P*<0.001 vs. INF; one-way ANOVA- SNK.

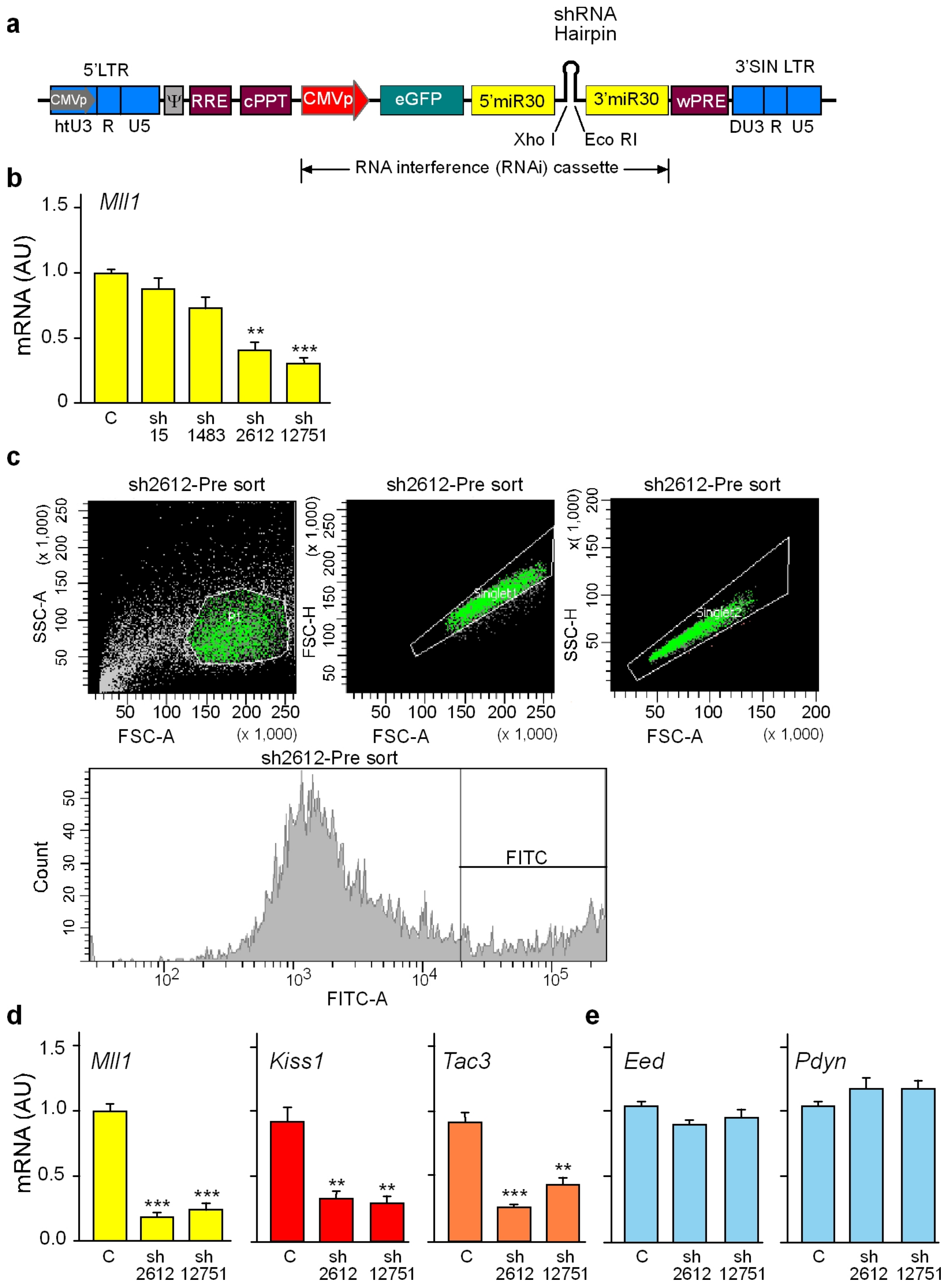

Supplementary Fig. 7: RNAi-mediated silencing of the *Mll1* gene. (**a**) Diagram of the pPRIME lentivirus delivery vector [1] used to target shRNAs to *Mll1* mRNA. The vector contains an artificial miR30 and replaceable hairpin structure able to accommodate up to three custom designed hairpins [2]; Xho I and Eco RI restriction enzyme sites are used as cloning sites to insert the

hairpin(s). The 5'LTR contains the heterologous U3 promoter (htU3), the repeat region/transcription initiation site (R), and the polyadenylation region (U5). The 3'LTR also contains R and U5 regions, but the 3'LTR U3 segment (DU3) bears a 400 bp deletion (SIN) that causes vector self-inactivation. The heterologous promoter htU3 is the cytomegalovirus promoter (CMVp); the CMVp also serves as the artificial miRNAi cassette promoter. The other components of this vector include the packaging signal (Ψ), the Rev response element binding site (RRE), the central polypurine tract (cPPT), and the woodchuck-hepatitis-virus posttranslational regulatory element (wPRE). A sequence encoding eGFP is located immediately upstream of the miR30 sequence and is transcribed under the control of the CMV promoter. The vector carrying different shRNAs targeting *Mll1* is termed LV-shMll1-GFP. **(b)** Effect of different LV-shMll1-GFP constructs on *Mll1* mRNA levels in R22 hypothalamic cells, three days after infection. (**c)** Gating conditions for detection of GFP positive cells by flow cytometry. Cell isolation was performed by fluorescent activated cell sorting (FACS) capturing the population of cells (P1) displaying >10^4 fluorescent units on the FITC channel. Purified sh2612- and sh12751-infected cells were expanded and used for subsequent experiments. (**d**) *Mll1, Kiss1 and Tac3* mRNA levels in cells sorted after transduction with sh2612, sh12751 or a control LV-pPRIME plasmid (C) carrying a mutated shRNA against firefly luciferase [1]. (**e**) *Eed* and *Pdyn* mRNA content in the same cells. RNA expression data were normalized by dividing each individual value by the average of the C group Bars are mean ± s.e.m. (n= 3) (** = P<0.01, and *** = P<0.001 vs. C group; one-way ANOVA-Dunnett's test.

Supplementary Table 1. Primers used

Primers for RT-qPCR

Species	*Gene*	*Accession #*	*Primers*	*Sequence*	*Amplicon (bp)*
RAT	*Cxxc1*	NM_001079698.1	rCxxc1-F-qPCR	GGTGCTCTGTGAGCGGAGAT	106
			rCxxc1-R-qPCR	GCAGTAGATGGGGGCGTTCT	
	Dpy30	NM_173117	rDpy30-f	AGTACGGGCTCACAGACAGC	165
			rDpy30-r	CCTTTGCGAGCACAGCAAGT	
	Eap1	NM_001012470.1	rEAP1-F	ACTGTATCCTCTGCTCCGTCATC	82
			rEAP1-R	ACCGATCCAGGCCTCTTACC	
	Eed	NM_1106278	rEed-RT-F	GAACAGCAACCCGGACCTCTCG	134
			rEed-RT-R	TTCCCTTTCCCCAGCTTTTCCTTC	
	Gpr54	AF115516.1	rGpr54-F	GTCGGGAACTCACTGGTCAT	115
			rGpr54-R	ACGCAGCACAGAAGGAAAGT	
	Grik5	NM_031508.2	rGrik5 mRNA F	CCAGCCCTCCGTCCCACCAG	86
			rGrik5 mRNA R	CCGGCATCTTCCTCCCCTCCTC	
	Grin2a	NM_012573.3	rGrin2a mRNA F	GGAACCCGCTAAACCTGGTG	113
			rGrin2a mRNA R	CCATGGTCGCCACTTAGGGT	
	Grin2d	NM_022797.1	rGrin2d mRNA F	AGCTCTGCGACCTGCTGT	190
			rGrin2d mRNA R	CCAAGCTGCAGGAAGGTGGA	
	Kiss1	NM_181692	rKiss1-RT-F	TGGTGAACCCTGAACCCACAGGC	136
			rKiss1-RT-R	CGGCGGGCATGGCGATGTT	
	Mkrn3	XM_218735.10	rMkrn3-F	AGAGGGAAGCGTGCTGTTA	205
			rMkrn3-R	AGCCTTAGCAGACCAGACCA	
	Mll1	XM_008766179.1	rMll1-F-qPCR	TAACGATGCTGGCCGTTTGC	198
			rMll1-R-qPCR	GCAGGATGTGAGGCAGCAAC	
	Mll2	XM_006257393.2	rMll2-F2	TCTTCCATTGAGACACTGGTGG	197
			rMll2-R2	ACACTGCGAACAGGCCAAGA	
	Mll3	XM_008762670.1	rMll3 F	GGAACTTTTGGCAGTGCCGC	197
			rMll3 R	GCTCACCAACACGCCAGCTT	
	Mll4	XM_008759254.1	rMll4-F qPCR	CTCCCAGAAGCTCCTTCCCG	171
			rMll4-R qPCR	TCTCAAAGCGCAGGTGTGGA	
	Nell2	NM_031070.2	rNell2 mRNA F	GCGTACGTGGATGGCAAGTG	181
			rNell2 mRNA R	GGCAATCTAAAGCCGGGCAG	
	pEnk	XM_006237835.1	rpEnk -F	CTCGTAGCGCTTGGGTCCTG	193
			rpEnk -R	GAACTCGGGCTTGGACACCT	
	Pdyn	XM_017591511.1	rPdyn-F	TCCCTGTGTGCAGTGAGGAC	118
			rPdyn-R	CAAAAGCCCCGGCATGTCTC	
	Ppia	M19533.1	rCycA F	GGCAAATGCTGGACCAAACACAA	222
			rCycA R	GGTAAAATGCCCGCAAGTCAAAGA	
	Set1a	XR_590498.1	rSeta-F-qPCR	CGGGTTGTAGAGCGGACCAT	118
			rSETa-R-qPCR	GTAGAGCGGACACGACCTCC	
	Set1b	XM_008760883.1	rSet1b-F-qPCR	GGAGGACGGTATCCGTGAGC	104
			rSet1b-R-qPCR	CTGGCACGGCTGCTATTGAG	
	Tac3	NM_019162.2	rTac3-F	GCAGGGTGGGAGGCTCAGTAAGG	193
			rTac3-R	TGCCCATAAGTCCCACAAAGAAGTCG	
	Tac3R	NM_017053.1	rTacR3-F	GGCTGCCCTATCACGTGTATTTCA	164
			rTacR3-R	AGCCTGCGCGGAATCTTTG	
	Ttf1	NM_013093.1	rTtf1-F	GCTGCCGCGGCCATCTCTG	233
			rTtf1-R	GGCGCGTCCCACATCTCACCA	
	Utx	XM_008773047.1	rUtx-F-qPCR	CCTCTGGACTTGCAGCACGA	144
			rUtx-R-qPCR	TGCCTGGAGGTAAGCTCTGC	
	Wdr5	XM_015149291	rWdr5 F	CGGATCCAGTCTCAGCCGTT	143
			rWdr5 R	GGACACCGGAGGATTGTCGT	
	Wdr82	XM_001073342	rWdr82-F-qPCR	CCGCGAGAACTCGGACAAGA	118
			rWdr82-R-qPCR	CTTTGGTTTGCCCTCCTGGC	
	DPY30	NM_001261093	mkyDpy30 F	ACTGGTATCCGGGGACTGTGACTTG	200
			mkyDpy30 R	GCACGAGTTGGCAAAGACTGGAGAT	

**Rhesus Monkey**	*GAPDH*	NM_001195426.1	mkyGAPDH F	AAGGGCATCCTGGGCTACA	68
			mkyGAPDH R	GAAGAGTGGGTGTCGCTGTTG	
	MLL1	XM_015115824	mkyMLL1-F	TGTTGCCCTTGGCCGAAAAC	189
			mkyMLL1-R	TGGGTGGAGCAAGAGGTTCA	
	SETD1A	XM_015125982	mkySETD1A-R	CACGAGTGGGGTCTGTTTGC	161
			mkySETD1A-F	GTGTCATGGTCCACCCGGAA	

Primers for ChIP-qPCR

Species	*Gene*	*Accession #*	*Primers*	*Sequence*	*Amplicon (bp)*
**RAT**	*Eed*	NM_1106278	rEed-CHIP F	AATAAAAGGCACGCGACCCCATAC	109
			rEed-CHIP R	TGCAAAAGAAAGTCGAGCCAATG	
	Kiss1	NM_181692.1	rKiss1-CHIP F	TCGGGCAGCCAGATAGAGGAAGC	91
			rKiss1-CHIP R	TTGAGGGCCGAGGGAGAAGAG	
			rKiss1 Site1-F	AGCCCCACACCCATCTCTGAAGT	142
			rKiss1 Site1-R	CTGGACCCGGATACGATGCGATGG	
			rKiss1 Site2-F	GGGGCAGTGGGGCCACCGCTGTA	94
			rKiss1 Site2-R	AACTCAGTCCTCCGGTACCTCCGCAAATCTGTG	
	Nell2	NM_031070.2	rNell2CHIP F	AGGCGGCTCCAAGTTCG	190
			rNell2CHIP R	TGGCGTGCATCGTCTCAG	
	Pdyn	NM_019374.3	rPdynCHIP F	CTGCCTTTCTCCTACTTTTGTCTCTGTTTT	109
			rPdynCHIP R	CGGGGGTGGATTCTCGGTGTAG	
	Tac3	NM_019162.2	rTAC2 CHIP-F	ACGTGCGTGTCTGGGTATGTGA	123
			rTAC2 CHIP-R	GGAGGGTTTGGGGGAGTCG	
	Ttf1	XM_006233882.3	rTtf1CHIP F	GTCTTGTTGCTTTAGCGCTTA	125
			rTtf1CHIP R	GTACACGTAACGGAGTGGAC	

Primers for FISH

Species	*Gene*	*Accession #*	*Primers*	*Sequence*	*Amplicon (bp)*
**RAT**	*Kiss1*	*NM_181692.1*	rKiss1-F	ATGATCTCGCTGGCTTCTTGGCAG	393
			rKiss1-R	TCAGCCCCGTGCCGCCCGCGC	
	Mll1	XM_008766179.1	rMll1 ISH -F2	TTAAACGGACCCCTTCTGCTATGT	522
			rMll1 ISH -R2	TCTATGAACCGTCGGGGAGTCTT	
	Mll2	XM_008765859.2	rMll2-F-ISH	AAGCCACCGGTGCTCTCTTT	439
			rMll2-R-ISH	TGGGAAGGTGTGGCTCAGTG	
	Mll3	XM_006235840.3	rMll3 ISH F	CGGCCCGTGTTTGTCATCAGG	425
			rMll3 ISH R	CGGTGCGTTCAGCTCTCCAGTG	
	Mll4	XM_008759254.2	rMll4 ISH F	ACCCCCACCTCCACCCCCACTA	630
			rMll4 ISH R	CCGCGCTCCACTCATTCCACTAAA	
	Set1b	XR_001836008.1	rSet1b-F-ISH	CGGTGCCCTCACCCCTATTT	535
			rSet1b-R-ISH	CCCACCATCCTGGCCTACTG	

22-mer oligodeoxynucleotides encoding shRNAs

Species	*Gene*	*Accession #*	*Primers*	*Sequence*
Rat	*Mll1*	XM_008766179	shrMLL1-15	CCGGGAACGTGTGTAATCGGCC
			shrMLL1-1483	TAGCAGTCCCAGTATCGATACC
			shrMLL1-2612	GAGCTGTCCAAAGATCGCGATA
			shrMLL1-12751	GAGGACACTCCTACTCGGTATA

Primers to detect shMLL1-mediated *MLL1* mRNA knock-down

Species	*Gene*	*Accession #*	*Primers*	*Sequence*	*Amplicon (bp)*
Rat	*Mll1*	XM_008766179	shMLL1 15-F	GCAGCCGCGCCGCACTGGAAGTG	190
			shMLL1 15-R	CCGCTCCGGGCTCCAGGGCTTCC	
			shMLL1 1483-F	CCCAGCACTCCTCTCAGATGTCTTCAG	199
			shMLL1 1483-R	GTAATGACAGGAGAGGCAGACGGTATTCG	
			shMLL1 2612-F	TACTTTTCCTTCTCACTCCCTAACTCAGTCTG	182

			shMLL1 2612-R	GCTCCTCCGGGGCTGTGTCTT	[illegible]
			shMLL1 12751-F	AGCGGCTTCCTCTCCATTCCTTCTCTTTATTC	193
			shMLL1 12751-R	GCTCCTGCCCACTTCCCCACCTG	

Oligodeoxynucleotides encoding sgRNAs targeting *Kiss1* enhancer Site1

Species	***Gene***	***Accession #***	***Primers***	***Sequence***	***Position upstream TSS***
**RAT**	*Kiss1*	JX139031	rKiss1enh g1-F	CACCGCCCTAGGAATAGAAGAGAGGAC	-3182
			rKiss1enh g1-R	AAACGTCCTCTCTTCTATTCCTAGGGC	
			rKiss1enh g2-F	CACCGCCCATGTGAGGTACTTCAGAG	-2716
			rKiss1enh g2-R	AAACCTCTGAAGTACCTCACATGGGC	
			rKiss1enh g3-F	CACCGTTCCAGCCCCCTCGCCAACAAT	-2433
			rKiss1enh g3-R	AAACATTGTTGGCGAGGGGGCTGGAAC	
			rKiss1enh g4-F	CACCGTCCCCACCCTGACTTTATGCAG	-2205
			rKiss1enh g4-R	AAACCTGCATAAAGTCAGGGTGGGGAC	
			rKiss1enh g5-F	CACCGTCCTACCATCCAGGACACGAGG	-1694
			rKiss1enh g5-R	AAACCCTCGTGTCCTGGATGGTAGGAC	

Primers for PCR-cloning of KRAB domain

KRAB F-infusion	AAAGAAAAAGGGATCCGGAGGGATGGATGCTAAG
KRAB R-infusion	CGTATGGGTAGGATCCGGGCTCTTCTCCCTTCTC

Primers to detect dCas9 mRNA

dCas9 P2 Fw	TTACCGCCCGGAAAGAGATTATTG
dCas9 P2 Rv	GCTCGGAGTTCAGATTGGTCAGTT

Supplementary Table 2. Promoters used in gene reporter assays

Gene Promoter	***Species***	***Accession #***	***Size (bp)***	***From****	***To****
Eap1	rat	NM_001012470.1	2556	-2345	+210
Eed	rat	NM_001106278.2	2107	-1850	+256
KISS1	human	NM_002256.3	1339	-1317	+22
MKRN3	human	NM_005664.3	439	-446	-7
Nell2	mouse	NM_001289653.1	881	-851	+30
PDYN	human	NM_001190892.1	790	-450	+340
PENK	human	NM_001135690.2	686	-479	+207
TAC3	human	NM_001178054.1	835	-766	+69
Ttf1	rat	XM_006233882.3	5181	-5180	+1

* With respect to the transcription start site (TSS)

Supplementary Table 3. Primary antibodies used

Target	*Host*	*Source*	*Catalog #*	*Use*
Beta-Galactosidase	Mouse	ICN Biomedicals	55976	ChIP
Beta-Galactosidase	Rabbit	Cortex Biochem	CR7001RP2	ChIP
EED	Mouse	Millipore	05-1320	ChIP
GFP	Goat	abcam	ab6673	IHC
H3K4me1	Rabbit	Active Motif	61633	ChIP
H3K4me2	Rabbit	abcam	ab7766	ChIP
H3K4me3	Rabbit	Active Motif	39159	ChIP
H3K9me3	Mouse	Active Motif	61013	ChIP
H3K27ac	Rabbit	Active Motif	39133	ChIP
H3K27me3	Rabbit	Active Motif	39155	ChIP
p300	Mouse	abcam	ab14984	ChIP
MLL1	Rabbit	Ali Shilatifard	569/Rb065	ChIP
MLL2	Rabbit	Ali Shilatifard	401/Rb9173	ChIP
MLL3	Rabbit	Ali Shilatifard	564	ChIP
MLL4	Rabbit	Ali Shilatifard	403/Rb823	ChIP

References

1. Stegmeier,F., Hu,G., Rickles,R.J., Hannon,G.J., & Elledge,S.J. A lentiviral microRNA-based system for single-copy polymerase II-regulated RNA interference in mammalian cells. *Proc. Natl. Acad. Sci. U. S. A* **102**, 13212-13217 (2005).

2. Sun,D., Melegari,M., Sridhar,S., Rogler,C.E., & Zhu,L. Multi-miRNA hairpin method that improves gene knockdown efficiency and provides linked multi-gene knockdown. *BioTechniques* **41**, 59-63 (2006).
